# DNA Barcoding studies on Thrips in India: Cryptic species and Species complexes

**DOI:** 10.1038/s41598-017-05112-7

**Published:** 2017-07-07

**Authors:** Kaomud Tyagi, Vikas Kumar, Devkant Singha, Kailash Chandra, Boni Amin Laskar, Shantanu Kundu, Rajasree Chakraborty, Sumantika Chatterjee

**Affiliations:** Centre for DNA Taxonomy, Molecular Systematics Division, Zoological Survey of India, M- Block, New Alipore, Kolkata, 700 053 West Bengal India

## Abstract

Thrips are one of the major sucking pest and vector of plant viruses causing huge economic loss in agriculture. The accurate identification of thrips is crucial for effective pest management strategies. However, morphology based identification has limitations and warrants integration of molecular data. We attempted the largest DNA barcoding initiative on 370 sequences of 89 thrips morphospecies including 104 novel sequences from 39 morphospecies, including the type specimens of four species. The results of multiple species delimitation methods (BIN, ABGD, GMYC and bPTP) were consistent for 73 species (82%) with their morphological identifications. A total of 107 molecular operational taxonomic units (MOTUs) was recovered for 89 morphospecies by superimposing multiple methods and applying a three level nomenclature system. We detected more than one MOTU in 14 morphospecies indicating to have cryptic diversity including, two major vector species (*Frankliniella schultzei* and *Thrips palmi*). However, four morphospecies (*Thrips moundi*, *Thrips carthami*, *Haplothrips andersi* and *Haplothrips gowdeyi*) showed low genetic distances between them with overlapping in barcode gap that requires further analysis with multiple molecular markers and more specimens from wide geographical areas for better taxonomic judgment. We also presented the advantage of simultaneous use of multiple delimitation methods for detection and identification of cryptic species.

## Introduction

Members of the insect order Thysanoptera with two recognized suborders, the Terebrantia and Tubulifera, are commonly called thrips. Thrips are minute insects, usually 1–3 mm in length and not readily noticeable. Typically the adults have four slender wings, each with a long fringe of marginal cilia, but the most remarkable feature of thrips is the asymmetry of their mouthparts, that is only the left mandible is present. Moreover, the larvae have a pair of tarsal claws, these are reduced in the adult to a pair of ensheathing spoon-shaped sclerites between which lies the bladder-like arolium^1^. These exopterygote (development of the wings outside the body) insects with holometabolous (complete metamorphosis) development exploit a wide range of habitats with diverse biologies, ranging from phytophagy, predation to fungus-feeding^2^. There are approximately 6102 described species of thrips globally, and about 1% of them have been reported as pollinators, predators, pests or vectors of plant viruses^3–5^.

Thrips are one of the major sucking pests, that can seriously hamper crop production by direct feeding damage and acting as vectors of Tospoviruses (genus Tospovirus, family Bunyaviridae). These are RNA viruses transmitted by thrips and are one of the most important plant viruses. Thrips are the sole transmitters of Tospoviruses, affecting plant species in several unrelated plant families across the globe^6^. Their ability to act as vectors for these viruses has severely hampered the agricultural economy over the past two decades. Though the actual data on the extent of damage is not available worldwide, around $50 million loss per year has been estimated by one thrips species, *Frankliniella occidentalis* in the Netherland^7^. The peculiarity in tospovirus transmission by thrips is that only larvae can acquire the viruses while adults can transmit^8^. Currently, 15 species in six genera (*Ceratothripoides*, *Dictyothrips*, *Frankliniella*, *Neohydatothrips*, *Scirtothrips* and *Thrips*) of subfamily Thripinae (Thripidae) have been reported as vectors for tospoviruses^6, 9^. Most of the pest species in the order Thysanoptera belongs to three genera, namely *Frankliniella*, *Thrips*, and *Scirtothrips*.

The erroneous identification of an economically important species may have parallel and serious ramifications as it will generate confusing data for other fields of biology^7, 10–12^. The precise identification of thrips species is the first and fundamental step to develop genetic and other biological information that is essential for effective management strategies. The identification of thrips species is primarily based on morphological characters such as color, chaetotaxy, body architecture, etc. However, their minute size, cryptic behavior, sexual dimorphism, high degree of similarity in various developmental stages and polymorphism (in color, wing development, body size, etc.)^1^ are some of the obstacles for morphology based identification.

Considering these difficulties, it is vital to use supporting methods to identify thrips species and resolve other taxonomic issues. Molecular tools have been found useful in the last decade to supplement various fields of biology ranging from systematics to ecology^13^. DNA Barcoding employs the partial fragments of mitochondrial cytochrome *c* oxidase I gene (mtCOI) for species-level identification, and this has gained wide acceptance as a supplementary method to resolve taxonomic ambiguities^14, 15^. Mitochondrial genes have also been used to estimate genetic diversity below species level^16^. Its utility as a rapid and authentic tool for species identification is well recognized in a wide variety of animal taxa across the globe (http://www.ibol.org/resources/). This technique has also been used in thrips identification^17–20^, detection of cryptic species^7^, invasive genetics^21–24^, population structure^25^, development of species-specific markers^26, 27^ and phylogenetic analysis^2^. However, these DNA barcoding studies for thrips identification^18–22^ were focused on a limited number of vector species. A recent DNA barcoding study on thrips from Pakistan^28^ included 43 morphologically identified species. Out of these 26 species were also sequenced in our study.

In this current study, we tried to address these major shortcoming in DNA barcoding studies on thrips, i) a limited number of species and ii) unavailability of voucher specimen, which is a fundamental necessity to link the barcode data with morphology to be accessed for future studies. Here, we aim to test the utility of DNA barcoding in species identification on a large dataset from morphologically identified thrips in India. We applied multiple species delimitation methods to analyze the barcode sequences for detection of cryptic species and resolving species complexes. Our study is the largest attempt on DNA barcoding of thrips involving 89 morphospecies including barcode data from the type specimens of four species. This study establishes a comprehensive DNA barcode library of thrips linked to voucher specimens for future taxonomic research.

## Material and Methods

### Specimen collection and morphospecies identification

A total of 336 specimens was collected from 78 locations in India (Fig. S1). Specimens were collected during 2011–2015 by beating methods in a white plastic tray and preserved in 95–100% ethanol and stored at −20 °C until DNA analysis. After non-destructive DNA isolation, specimens were retrieved and mounted in Canada balsam onto glass slides for morphological work. The glass mounted specimens were identified with standard morphological keys^1, 29–34^ by the first author. The photographs were taken through a Leica Trinocular Microscope (Leica DM-1000) and using a Leica software application suite (LAS EZ 2.1.0). All voucher specimens are deposited in the National Zoological Collections (NZC) at the Zoological Survey of India Kolkata (Fig. S2, Table S1).

### DNA extraction, Polymerase Chain Reaction and Sequencing

The total genomic DNA was extracted from individual thrips specimens with a non-destructive method^2^ using QIAamp DNA Mini Kit (Qiagen, Valencia, CA). Briefly, an intersegmental abdominal cut was made to each specimen, and these were lysed overnight at 56 °C in buffer ATL with proteinase K. Qubit fluorometer (Life Technologies, USA) was used to estimate the DNA quantity and the extracted DNA was stored at −20 °C for downstream analysis. The Polymerase chain reaction (PCR) was performed by using 10 ng genomic DNA to amplify about 648 base pairs (bp) from the 5′ end of the mtCOI gene using primer pair LCO-HCO^35^. The PCR reaction was set in a 50 µl total volume containing 20 Picomoles of each primer, 20 mM Tris-HCl (pH 8.0), 100 mM KCl, 0.1 mM EDTA,1 mM DTT, 1.8 mM MgCl2, 0.25 mM of each dNTP, and 1U of Taq polymerase (Takara BIO Inc., Japan) with the following cycling parameters: 5 min at 94 °C; followed by 40 cycles of 30 s at 94 °C, 40 s at 49 °C, 1 min at 72 °C and final extension for 5 min at 72 °C. The amplified PCR products were checked in 1.2% agarose gel. The PCR amplified products were purified using the QIAquick Gel Extraction Kit (Qiagen, Valencia, CA) following the manufacturer’s protocols. Approximately, 15 ng of the purified PCR product was used for sequencing. The cycle sequencing was performed with BigDye®Terminator ver. 3.1 Cycle Sequencing Kit (Applied Biosystems, Inc.) using 3.2 Picomoles of both forward and reverse PCR primers on a ABI thermal cycler with following parameters: 96 °C for 1 min, then followed by 25 cycles of 96 °C for 10 s, 50 °C for 5 s and a final extension at 60 °C for 1 min 15 s. The cycle sequencing products were cleaned by using BigDye X-terminator kit (Applied Biosystems Inc.) and loaded on 48 capillary ABI 3730 Genetic analyzer, housed at the Zoological Survey of India, Kolkata.

### Sequence analysis, genetic distance and haplotyping

A total of 336 sequences for 87 morphospecies were generated in the current study (Table S1). The generated sequences were aligned with the 34 representative barcode sequences pertaining to eight morphospecies from India generated in a previous study^36^ (Table S2). Hence, the final dataset comprises 370 sequences of 89 morphospecies. The SeqScape software version 2.7 (Applied Biosystems Inc.) was used to analyze the forward and reverse chromatograms to obtain the consensus sequences. The generated sequences were submitted to GenBank and BOLD database under the project ‘DNA barcoding thrips of India’ for acquiring accession numbers and BOLD-IDs. We employed Kimura-2 parameter (K2P) model to calculate intra and interspecific pairwise genetic distances as it is computationally fast and the most widely used distance measure method (www.bold.org). However, the use of the K2P distances in DNA barcode analysis has been challenged^37, 38^ and patristic distances (path length between tips in the most likely topology) approaches have been proposed for species delimitation as these justify better for multiple substitutions at the same nucleotide site^39, 40^. Hence, patristic distances were also estimated to compare with the K2P distances. The K2P distances were estimated using MEGA6.0^41^, and patristic distances were calculated using the program PATRISTICv1.0^42^ from ML tree (GTR+G+I) and presented in a boxplot created online using BoxPlotR: a web-tool for generation of box plots (http://shiny.chemgrid.org/boxplotr/). Haplotype data were generated in DnaSP5.10^43^ to identify the unique haplotypes.

### Barcode Tree analysis, Species delimitation and MOTUs nomenclature

Two tree building methods, Neighbor-Joining (NJ) and Bayesian analysis (BA) were implemented to test the reciprocal monophyletic criteria for species delimitation. The NJ tree was constructed using MEGA6.0 with Kimura-2 parameter (K2P) model as suggested in barcoding studies. The bootstrap support for a NJ tree for the branch nodes was estimated through bootstrapping with 1000 replicates. The model selection was based on the Bayesian Information Criterion (BIC) computed by PartitionFinder version 1.1.1^44^. The Partitionfinder resolved the same model (GTR+I+G) for all three codon positions; hence we did not further partition the data. The best scheme was chosen based on the score of lnL: −26777.63144 and BIC: 58402.6989637. The model GTR+I+G (NST = 6) was selected and Mr. Bayes 3.1.2^45^ was used for Bayesian analysis on the full dataset with 370 sequences and additionally with 138 unique sequences for haplotypes. The Bayesian analysis with the metropolis-coupled Markov Chain Monte Carlo (MCMC) was run for 50,000,000 generations with trees, saving at every 100^th^ generation (the first 25% of samples were discarded as burn-in). The Convergence metrics were generated by MCMC analysis, checked as standard deviation (SD) of split frequencies reached under 0.01 and the potential scale reduction factor (PSRF) for all parameters approached 1.0. The trees generated through this process were represented using the online utility iTOL^46^.

To estimate the number of molecular operational taxonomic units (MOTUs) from the thrips dataset, we applied four methods: Automatic Barcode Gap Discovery (ABGD)^47^, the General Mixed Yule-coalescent (GMYC)^48^, Poisson-Tree-Processes (bPTP)^49^ and Barcode Index Numbers (BINs)^50^. The ABGD and BINs use clustering approaches based on genetic distance for MOTUs picking, while the GMYC and bPTP are coalescent-based species delimitation methods which execute model based approaches on gene trees with likelihood and Bayesian methods to find out MOTUs. These MOTUs picking methods implement different strategies and either underestimate or overestimate the taxa numbers. It is impossible to favor one method over another, and it is suggested to apply a wide range of species delimitation methods and choose the congruent results^51, 52^. Hence, we applied these different methods to compare the MOTUs number with our morphospecies and to verify the presence of cryptic species. BINs were assigned automatically on BOLD workbenchv3.6 (http://www.boldsystems.org; analyzes performed on 23 August 2016). The ABGD analysis was conducted on the web interface (www.abi.snv.jussieu.fr/public/abgd/) with the default settings, by K2P, Jukes-Cantor (JC69) and p distance model with relative gap width (X = 1.5). The GMYC and bPTP analysis were conducted on the species delimitation web server (http://species.h-its.org/). For GMYC, we first created a haplotype data file in DNaSP, which was used to generate an ultrametric tree in BEAST^53^ with the following settings: Yule model, relaxed lognormal clock, GTR+I+G model. The analysis was run for 50 million generations, with a sampling frequency of every 100 generations. The output trees were analyzed in Tree Annotator^53^ with following settings: 10% burn-in, 0.5 posterior probability limits, and the node heights of the target tree. For bPTP, a Maximum Likelihood (ML) tree was generated from haplotype data in RAXML-VI-HPC^54^ and then used it with the default setting at the species delimitation web server (http://species.h-its.org/ptp/). In both the analyzes, we removed the out groups before generating the haplotype data file.

Further, we applied a three-level nomenclature system for naming the MOTUs of all the studied species (Table S3). This nomenclature is based on a three-level hierarchical system (e.g., “Ia1”, meaning: MOTU level-1 “I” includes MOTU level-2 “a” includes MOTU level-3 “1”). MOTU level-1 represents the highest intra-morphospecific level defined as a monophyletic cluster of intermediate-level MOTUs. MOTU level-2 is the intermediate level most likely represents the level of biological species. MOTU level-3 is the lowest level corresponds to the basegroup and represents the genetic variability potentially observable at the level of a single individual^51^. This nomenclature of these MOTUs will set up a strong framework to study whether these MOTUs will gradually differentiate into a set of metapopulations into distinct morphospecies.

## Results

### Morphological identification

Morphological examination of the 336 specimens collected in this study identified 87 morphospecies in 52 genera in four families; Stenurothripidae, Aeolothripidae, Thripidae, and Phlaeothripidae (Fig. S2). Further, the specimens of Thripidae were categorized under three subfamilies: Panchaetothripinae, Sericothripinae, and Thripinae. One specimen of this dataset could be identified only to family level and one specimen each of *Dyothrips* sp., *Scirtothrips* sp. and *Streothrips* sp. probably represents new species which require more specimens for formal description. The study includes four species: *Neohydatothrips chandrai*, *Neohydatothrips plumeria*, *Taeniothrips bharokariiensis*, and *Thrips moundi* which were recently described from India^17, 33, 34^. Further, four invasive species, *Helionothrips aino*, *Parabaliothrips takahashii*, *Scirtothrips kenyensis* and *Thrips parvispinus* ^23, 55^ were also included here.

### DNA barcode based identification

**Figure 1:**
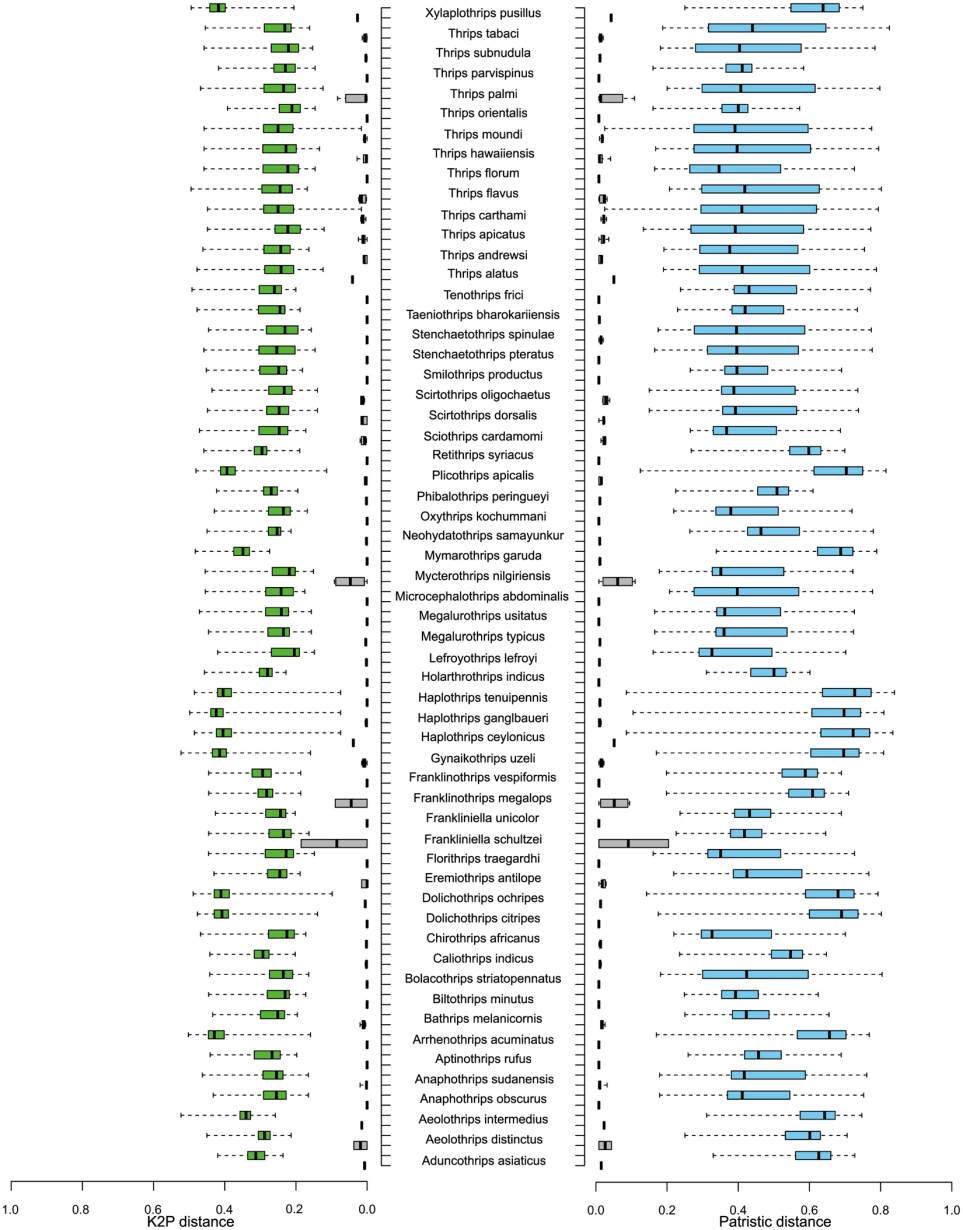
DNA barcoding gaps for thrips species based on the K2P and patristic distances. Center lines show the medians; whiskers extend to minimum and maximum values. Grey color: intraspecific distance; Sky blue (Patristic) and green (K2P): interspecific distance. Singleton species are excluded from the data analysis.

A total of 370 mtCOI sequences representing 89 morphospecies was analyzed in the current study. Haplotype data analysis detected 138 unique haplotypes in the complete thrips dataset (Fig. S3). The analysis revealed that overall K2P mean genetic distance (MGD) of the dataset was 0.2524. The MGD was increased hierarchically from within species (mean = 0.0101, standard error [SE] = 0.002), to within congeners (mean = 0.1871, SE = 0.0066), to within families (mean = 0.2362, SE = 0.010) in the K2P model (Table S4). The intra and interspecific MGD in three families (Aeolothripidae, Phlaeothripidae, and Thripidae) are provided (Table 1). Overall, the K2P genetic distance among the congeneric species was an average of approximately 18 times greater than that among individuals of the same species. The intraspecific distance showed substantial variations and ranged from 0 to 0.1830, with a mean value of 0.0101. Therefore, it is clear that a distinct barcode gap (lowest interspecific distance > highest intraspecific distance) exists in the current study. Patristic distances analysis resulted in almost the same data for intra and interspecific distances as compared with K2P (Fig. 1).

**Table 1:** Mean K2P genetic distance of different families under study.

Family	Number of genera (species)	Intraspecific		Interspecific	
		Min	Max	Min	Max
Aeolothripidae	5 (7)	0.00	0.046	0.1841	0.3693
Thripidae	37 (63)	0.00	0.1015	0.0194	0.3455
Phlaeothripidae	10 (18)	0.00	0.043	0.0770	0.2780

The family Stenurothripidae is not included here as it is represented by one species.

### NJ and BA tree analysis

**Figure 2:**
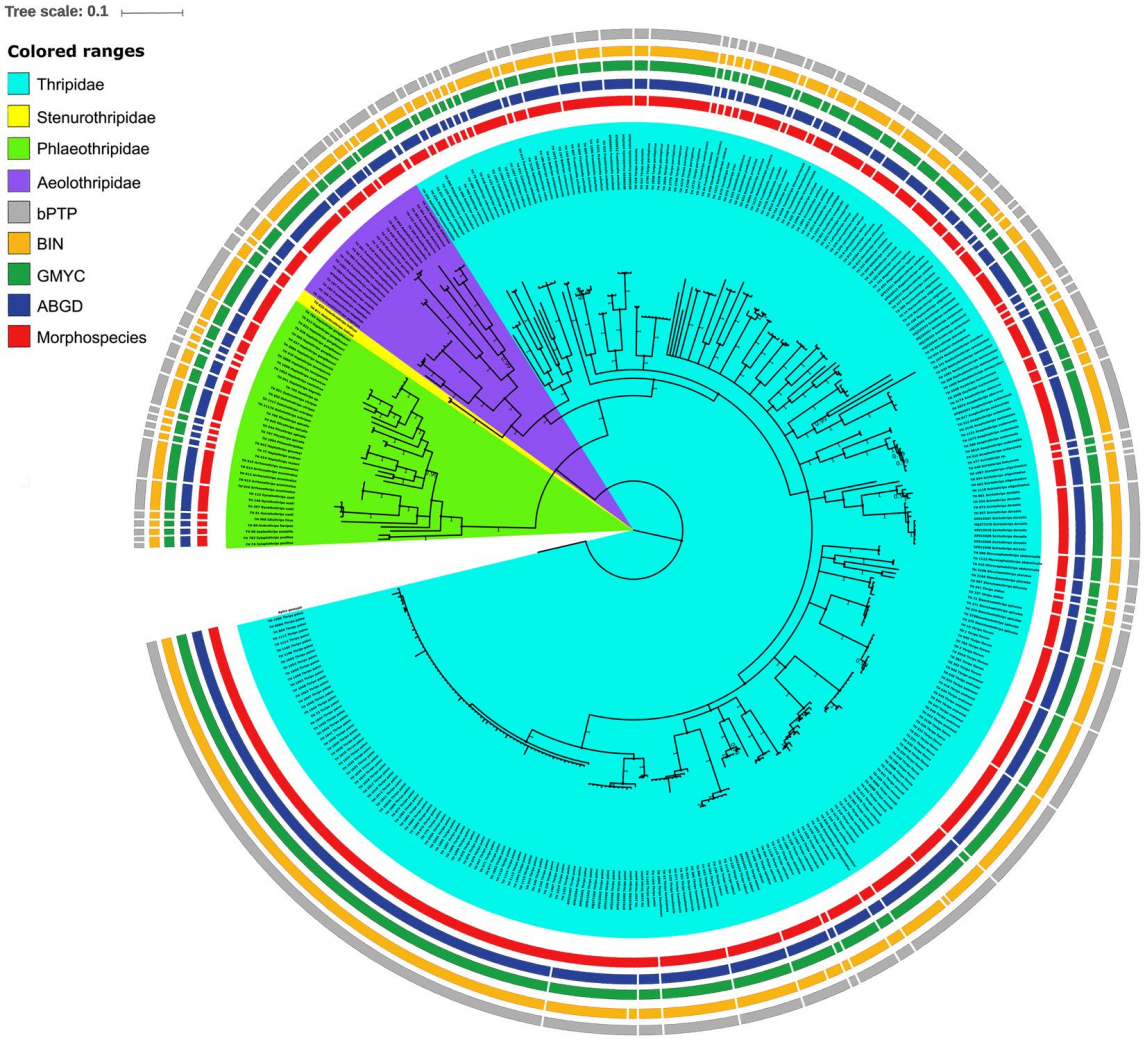
Bayesian inference gene tree with delineated MOTUs with posterior probability of the studied 89 thrips species. The four families: Phlaeothripidae, Thripidae, Stenurothripidae and Aeolothripidae were highlighted by color corresponding to the clade. Color bars indicate delineated MOTUs by different methods (Morpho species, ABGD, GMYC, BIN, and bPTP).

Both the NJ and BA tree building methods yielded the same topology representing four families Aeolothripidae, Phlaeothripidae, Stenurothripidae, and Thripidae (Figs 2, S4). The NJ tree (bootstrap support ≥ 99) and BA tree (posterior probability ≥ 0.99) showed that sequence records for 73 species (82%) of the 89 morphospecies formed distinct species clades as per our morphological identification. Analysis of the remaining 16 species showed i) two clades each in 10 species, three clades in *F. schultzei* and four clades in *T. palmi* indicating presence of cryptic diversity ii) DNA barcode gap overlapping between four distinct morphospecies in two genera: *Thrips* (*T. moundi* and *T. carthami*) and *Haplothrips* (*H*. *andresi* and *H*. *gowdeyi*). Further, three species, *Thrips hawaiiensis*, *Thrips florum*, and *T. andrewsi* under the ‘*hawaiiensis* species complex’ formed three separate clades with high support values.

### MOTUs estimation and Nomenclature

We used four species delimitation methods (BIN, ABGD, GMYC, and bPTP) in this study. All these methods have yielded almost identical results in the dataset and demarcated 97, 94, 96, and 99 MOTUs respectively. Therefore, a total of 107 MOTUs was recovered by superimposing all the four methods.

ABGD categorized the sequences into MOTUs based on the barcode gap if the genetic distance among specimens of the same species is smaller than the distance among specimens from different species. ABGD analysis for MOTUs detection was estimated with JC69, K2P and p distance criteria. Initial partition in all three criteria produced 94 MOTUs with P = 0.0215–0.0599, 0.0215–0.0599 and 0.0129–0.0599 respectively (Table S5). This method detected one MOTU for each morphospecies except in the case of four species where it gives more than one MOTUs. *F. schultzei* and *Thrips palmi* were detected with three MOTUs each by ABGD analysis. Two MOTUs were detected in *Mycterothrips nilgiriensis* and *Franklinothrips megalops* each. For these additional MOTUs, the intraspecific K2P and patristic distances were considerably higher than the average intraspecific distances of the dataset indicating that these additional MOTUs are more likely to correspond to genuine cryptic species. Further, two morphospecies, *T. moundi* and *T. carthami* were recovered as one MOTU by ABGD.

GMYC delimits species by a likelihood method using intra and interspecies branching models to reconstruct molecular trees. In the ultrametric tree, GMYC model likelihood value was higher than that of the null model indicating the presence of several biological species in the dataset. The GMYC analysis with single and multi-threshold models produced different results with 96 (confidence interval 93-107) and 87 MOTUs (confidence interval 85-93) respectively (Table S6). We chose the 96 MOTUs result as it was close to the results in the other methods. This method produced more than one MOTU in four morphospecies (*T. palmi*: 3, *F. schultzei*; 3, *M. nilgiriensis*: 2, and *F. megalops*: 2) as detected in the ABGD method. Two additional morphospecies, *Thrips andrewsi* and *Thrips alatus* each were recovered as two MOTUs. The morphospecies *T. alatus* is represented by two specimens with two haplotypes and the K2P and patristic distance between these two is significantly higher; indicating that these MOTUs correspond to genuine cryptic species. *T. andrewsi* was detected with two MOTUs and a low K2P and patristic distance between them. Further, *T. moundi* and *T. carthami* which were recovered as single MOTU by ABGD are grouped into two MOTUs by GMYC. However, only one sequence of *T. moundi* was included in one MOTU and the other five sequences of *T. moundi* are grouped with *T. carthami* sequences in the second MOTU. Two well established morphospecies, *Haplothrips gowdeyi* and *Haplothrips andresi* were grouped as single MOTU; these two species have low interspecific distances (K2P: 0.056 and patristic 0.054) without any haplotype sharing between them.

The bPTP is an updated version of the original PTP model which also adds Bayesian support on the input tree. It uses coalescence theory and assumes that intra- and interspecific substitutions follow two distinct Poisson processes and that intraspecific substitutions are significantly fewer than interspecific substitutions^50^. The bPTP analysis has estimated 99 MOTUs in the studied dataset. These methods produced additional MOTUs in nine morphospecies. In four species (*T. palmi*: 3, *F. schultzei*: 3, *M. nilgiriensis*: 2, and *F. megalops*: 2) the MOTUs recovery was similar to the ABGD and GMYC methods. *T. alatus* was split into two MOTUs by this method as recovered in the GMYC. Four morphospecies, *Aeolothrips distinctus*, *Aeolothrips intermedius*, *Scirtothrips oligochaetus*, and *Xylaplothrips pusillus* were split into two MOTUs each in the bPTP analysis; out of these four, in *A. intermedius* the intraspecific distances (K2P and patristic) were quite low. Further, *T. moundi* and *T. carthami* were recovered as single MOTU in this method as detected by ABGD (Fig. 2).

BIN analysis of 336 sequences recovered 95 MOTUs (Table S7). The sequences of two species which were obtained from the GenBank dataset were not included in the MOTUs (BIN) analysis at BOLD. If we effectively consider them as two MOTUs then the final MOTUs number will be 97. Here, split of three morphospecies (*F. schultzei*; 3, *M. nilgiriensis*: 2, and *F. megalops*: 2) into MOTUs was consistent with ABGD, GMYC, and bPTP methods. BIN produced four MOTUs in *Thrips palmi* compared to three MOTUs by all other methods. This extra MOTU for one sequence (TH-1037) which had a shared haplotype; so may not indicate to represent a true cryptic species. The morphospecies *A. distinctus* was recovered as two MOTUs similar only to bPTP results. Two morphospecies, *Thrips apicatus* and *Haplothrips ceylonicus* were recovered as two MOTUs each in this method exclusively. However, low distances (K2P and patristic) in *T. apicatus* and sharing of haplotypes in *H*. *ceylonicus* does not indicate the presence of cryptic species. Further, *T. moundi* and *T. carthami* is recovered as three MOTUs; six sequences of *T. moundi* were recovered as single MOTU and seven sequences of *T. carthami* were split into two MOTUs with one and six sequences respectively.

### Cryptic species detection

In total, 14 species were detected with more than one MOTU (Table 2). Among them, 12 species are likely to represent cryptic species; however, we have chosen only those species for possible cryptic events where more than one MOTU was detected by at least two delimitation methods. By applying these criteria, we recovered only six morphospecies (*A. distinctus*, *F. schultzei*, *F. megalops*, *M. nilgiriensis*, *T. alatus*, and *T. palmi*) as candidates for cryptic diversity (Fig. 2).

**Table 2:** Number of Molecular Operational Taxonomic Units (MOTUs) in 14 morphospecies detected by multiple automatic species delimitation methods and three-level nomenclature.

Sl. No.	Morphospecies	MOTUs estimation				Three-level Nomenclature
		ABGD	GMYC	bPTP	BIN	
1	*Frankliniella schultzei*	3	3	3	3	Ia1
						IIa1
						IIIa1
2	*Franklinothrips megalops*	2	2	2	2	Ia1
						IIa1
3	*Aeolothrips intermedius*	1	1	2	1	Ia1
						IIa1
4	*Aeolothrips distinctus*	1	1	2	2	Ia1
						IIa1
5	*Thrips palmi*	3	3	3	4	Ia1
						IIa1
						Ib1
						Ib2
6	*Thrips alatus*	1	2	2	1	Ia1
						IIa1
7	*Thrips apicatus*	1	1	1	2	Ia1
						IIa1
8	*Thrips andrewsi*	1	2	1	1	Ia1
						IIa1
9	*Thrips moundi*	1	2	1	1	Ia1
						Ia2
10	*Thrips carthami*	1	1	1	2	Ia1
						Ia2
11	*Mycterothrips nilgiriensis*	2	2	2	2	Ia1
						IIa1
12	*Scirtothrips oligochaetus*	1	1	1	1	Ia1
						IIa1
13	*Xylaplothrips pusillus*	1	1	1	1	Ia1
						IIa1
14	*Haplothrips ceylonicus*	1	1	1	2	Ia1
						IIa1

**Figure 3:**
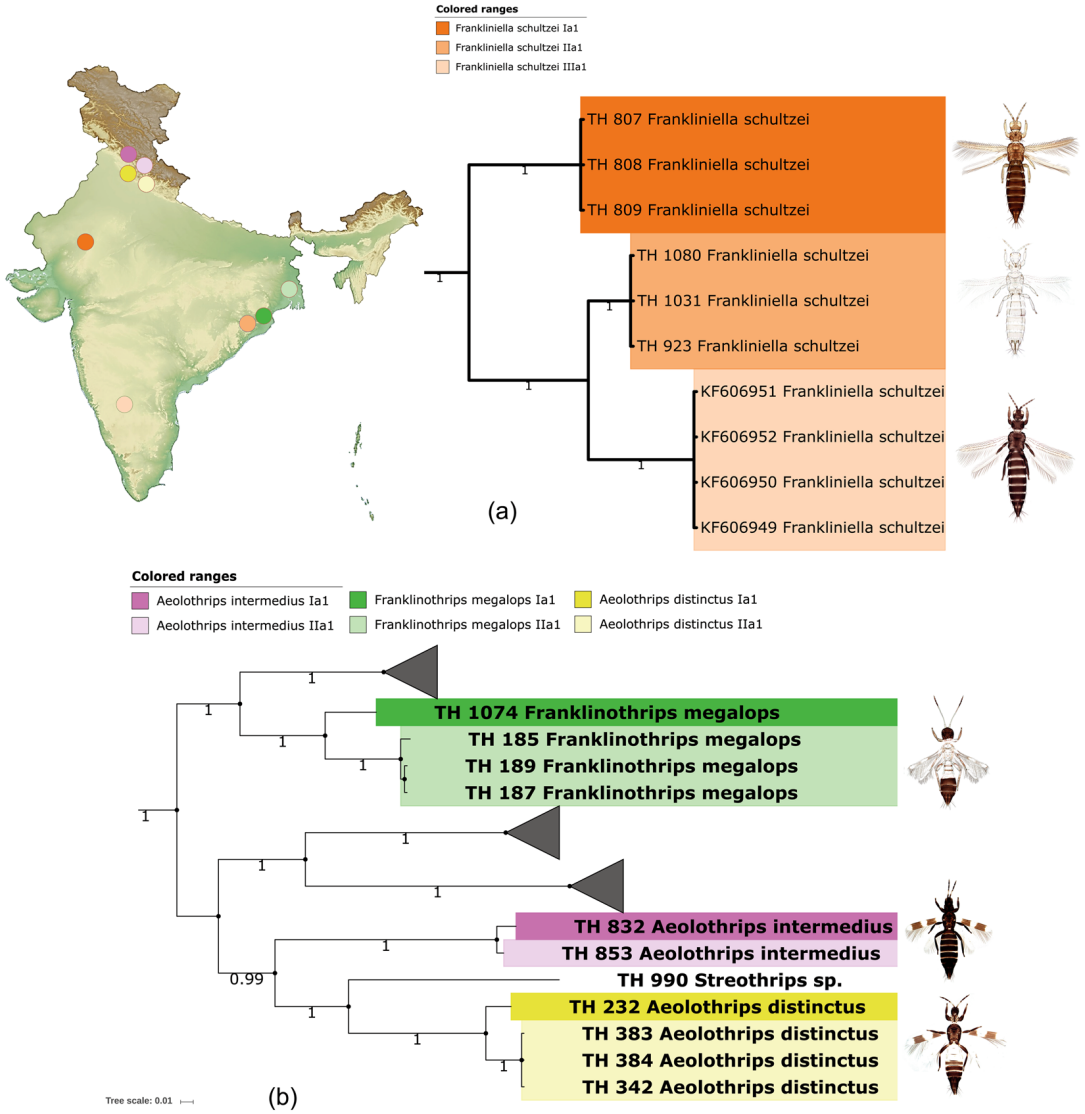
Pruned BA tree showing cryptic diversity of (**a**) *Frankliniella schultzei* and (**b**) *Franklinothrips megalops*, *Aeolothrips intermedius*, *Aeolothrips distinctus* with MOTUs nomenclature and geographical locations. Photographs used here were taken by the first author (K. T.). The original template of the topographic map used here is copied under the following attribution: created by Yug (Own work) [CC BY-SA 3.0 (http://creativecommons.org/licenses/by-sa/3.0)], via Wikimedia Commons; file URL: https://upload.wikimedia.org/wikipedia/commons/b/b9/Wikimaps_atlas-India-topographic_map-color-blank.jpg; page URL: https://commons.wikimedia.org/wiki/File%3AWikimaps_atlas-India-topographic_map-color-blank.jpg.

Three morphospecies of genus *Frankliniella* were analyzed in this study. Analysis revealed three allopatric cryptic species in one morphospecies, *F. schultzei*, of which 10 specimens formed three distinct clades in NJ and BA trees. These three clades corresponded to three MOTUs; *F. schultzei* Ia1, *F. schultzei* IIa1, and *F. schultzei* IIIa1 as recovered in all the four delimitation methods (Fig. 3a). These three MOTUs represent three different geographical locations from India with three unique haplotypes. The intraclade patristic distances of all clades were 0.0 and patristic distance between three clades was significantly higher ranging from 0.0827 to 0.1973. The same pattern is observed in K2P distances also. Morphologically, *F. schultzei* shows variation in body color, but we could not find any other morphological difference between the specimens of these three clades.

The morphospecies, *F. megalops* is represented by four specimens collected from two close locations and showed two cryptic species. Two MOTUs, *F. megalops* Ia1 with one specimen and *F. megalops* IIa1 with three specimens respectively were detected by all the four automated delimitation methods supported by two clades in NJ and BA trees. Specimens under *F. megalops* IIa1 revealed low patristic distance among them (0 to 0.005) which was significantly lower than the distance between these two MOTUs (0.0820 to 0.0860). Each of these MOTUs was represented by unique haplotype (Fig. 3b).

*Aeolothrips distinctus* with four specimens showed two cryptic species represented by two MOTUs, *A*. *distinctus* Ia1 with one specimen and *A*. *distinctus* IIa1 with three specimens. These were distinguished by all four automatic methods supported by two clades in NJ and BA trees. Specimens under *A*. *distinctus* IIa1 revealed low patristic distance (0.00) among them; which is significantly lower than the distance between these two MOTUs (0.036). These two MOTUs were represented by one haplotype each (Fig. 3b). The specimens of these two MOTUs were collected from very closely placed locations in Indian Himalayas.

**Figure 4:**
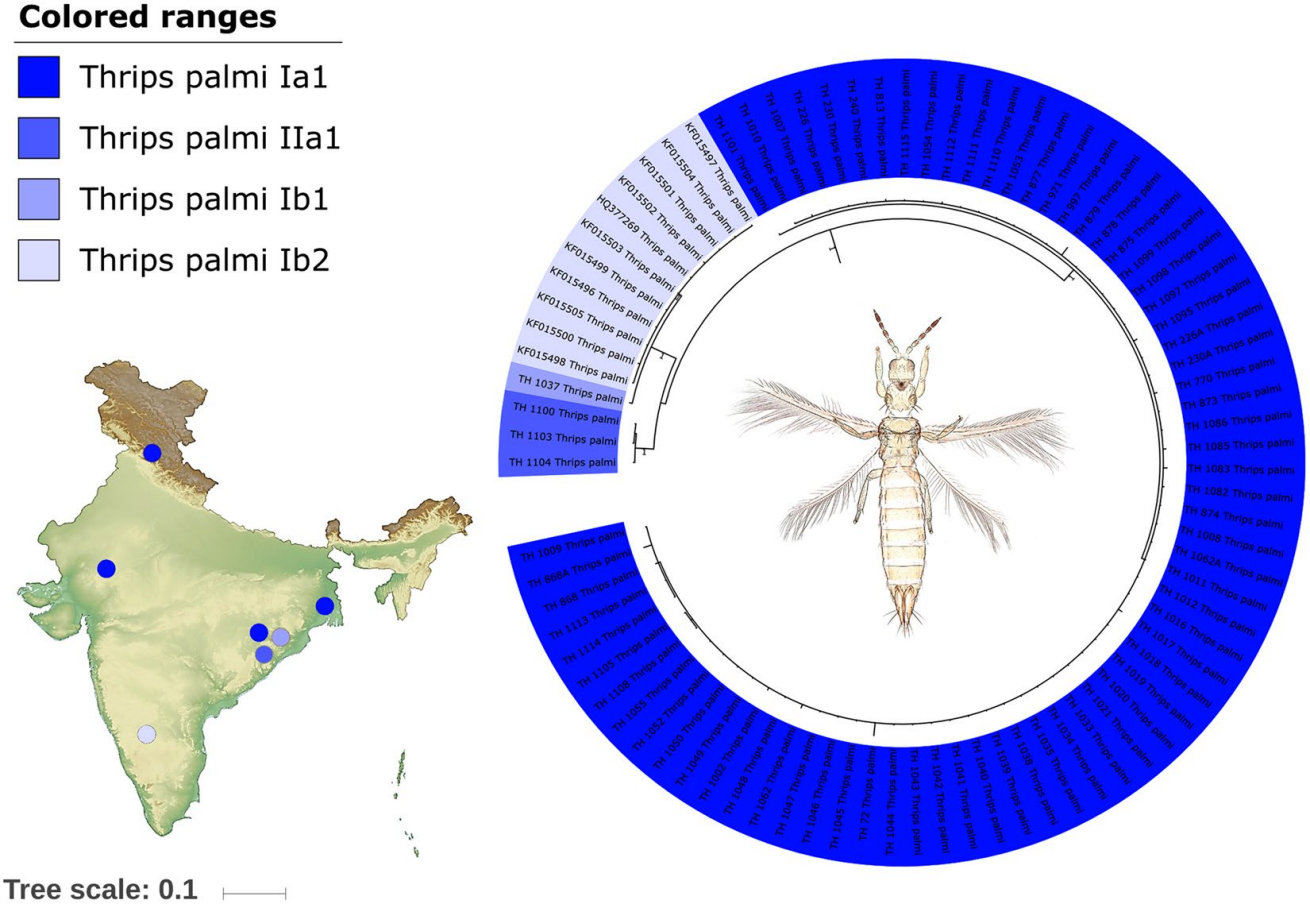
Pruned BA tree showing cryptic diversity of *Thrips palmi* with MOTUs nomenclature and geographical locations. Photograph used here was taken by the first author (K. T.). The original template of the topographic map used here is copied under the following attribution: created by Yug (Own work) [CC BY-SA 3.0 (http://creativecommons.org/licenses/by-sa/3.0)], via Wikimedia Commons; file URL: https://upload.wikimedia.org/wikipedia/commons/b/b9/Wikimaps_atlas-India-topographic_map-color-blank.jpg; page URL: https://commons.wikimedia.org/wiki/File%3AWikimaps_atlas-India-topographic_map-color-blank.jpg.

Analysis of 85 *T. palmi* specimens resulted in eight haplotypes (H9-H10, H126-H131) forming three distinct clades in both the NJ and BA tree. These three clades were also represented by three MOTUs (*T. palmi* Ia1, *T. plami* IIa1, and *T. palmi* Ib2) in ABGD, GMYC, and bPTP analysis (Fig. 4). BIN analysis showed an additional MOTU (*T. palmi* Ib1) with a single specimen (TH-1037). We observed low patristic distance among sequences of each of these MOTUs: *T. palmi* Ia1 is represented by 70 specimens (0.005 to 0.009); *T. palmi* Ib1 with 11 specimens, *T. palmi* Ib2 with one specimen (0 to 0.009), and *T. palmi* IIa1 with three specimens (0 to 0.015). The same pattern was observed in K2P distances also. Further, *T. palmi* Ib1 shows two haplotypes (H9 and H10) which share H9 with *T. palmi* Ib2. *T. palmi* IIa1 shows one haplotype (H128) from eastern India and *T. palmi* Ia1 shows five haplotypes.

**Figure 5:**
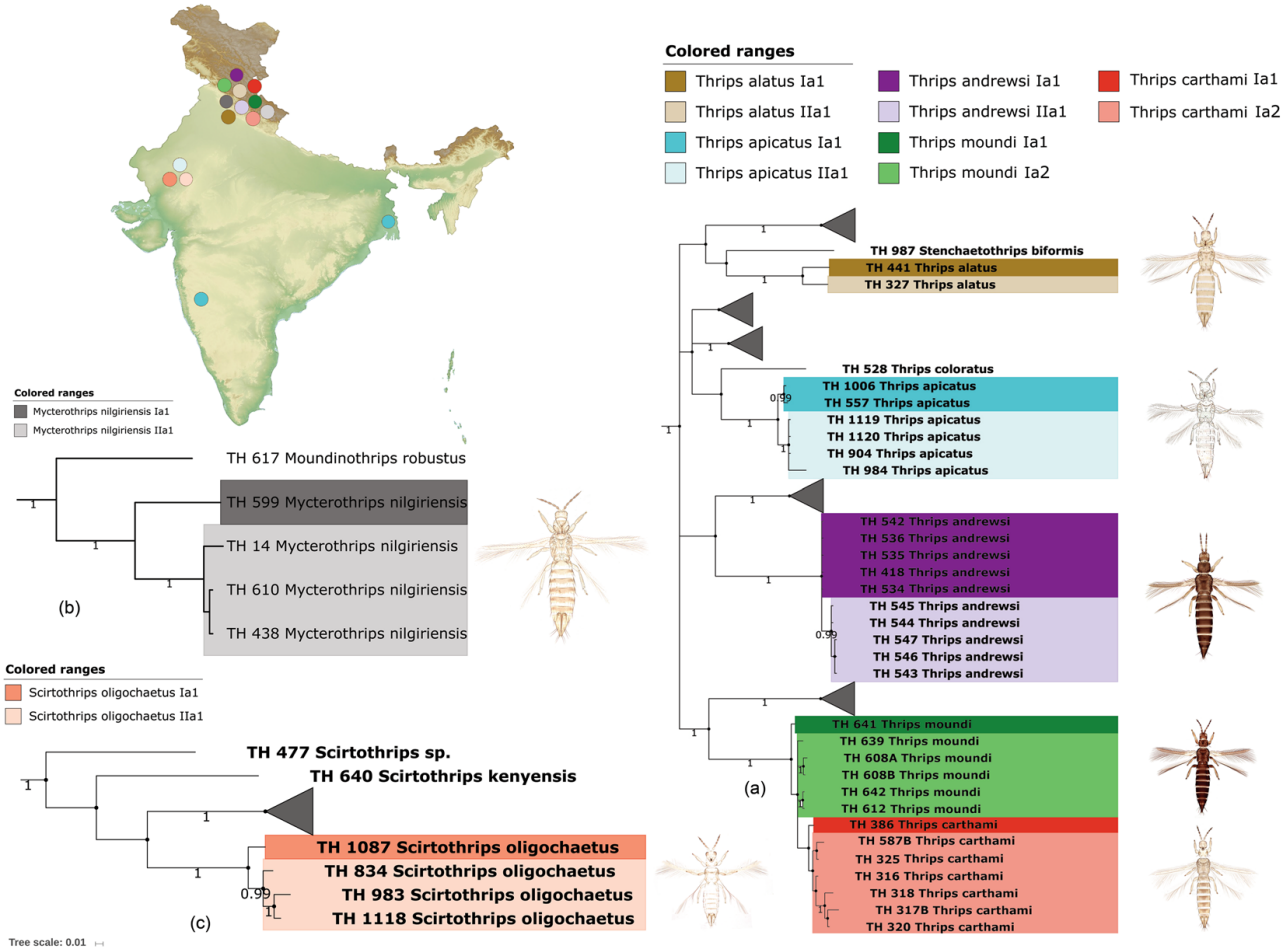
Pruned BA tree showing cryptic diversity of (**a**) Thrips alatus, Thrips apicatus, Thrips andrewsi, Thrips moundi, Thrips carthami (**b**) Myctrothrips nilgiriensis, and (**c**) Scirtothrips oligochaetus with MOTUs nomenclature and geographical locations. Photographs used here were taken by the first author (K. T.). The original template of the topographic map used here is copied under the following attribution: created by Yug (Own work) [CC BY-SA 3.0 (http://creativecommons.org/licenses/by-sa/3.0)], via Wikimedia Commons; file URL: https://upload.wikimedia.org/wikipedia/commons/b/b9/Wikimaps_atlas-India-topographic_map-color-blank.jpg; page URL: https://commons.wikimedia.org/wiki/File%3AWikimaps_atlas-India-topographic_map-color-blank.jpg.

*Thrips alatus* is restricted to Himalayas, and is closely related to *T. palmi*. *Thrips alatus* was depicted with two MOTUs (*T*. *alatus* Ia1 and *T*. *alatus* IIa1) with one specimen each and one haplotype in each MOTU (Fig. 5a). The patristic distance between these two MOTUs was 0.0423. These two specimens were collected from two close locations in Indian Himalayas. Both the NJ and BA analysis and two automatic species delimitation methods (GMYC and bPTP) have confirmed the presence of two MOTUs in this morphospecies. However, the genuineness of these two MOTUs to be considered as cryptic species needs more specimens.

The morphospecies *M. nilgiriensis* was represented by two cryptic species, each with one MOTU, *M. nilgiriensis* Ia1 with one specimen and *M. nilgiriensis* IIa1 with three specimens. These two MOTUs were distinguished by all four automated delimitation methods supported by two clades in NJ and BA trees. Specimens of *M. nilgiriensis* IIa1 revealed high patristic distance (0.011) among them; this was significantly lower than the distance between these two MOTUs (0.0941 to 0.102). Further, two haplotypes were detected corresponding to two MOTUs. All of these four specimens were collected from the same location in the Indian Himalayas (Fig. 5b).

**Figure 6:**
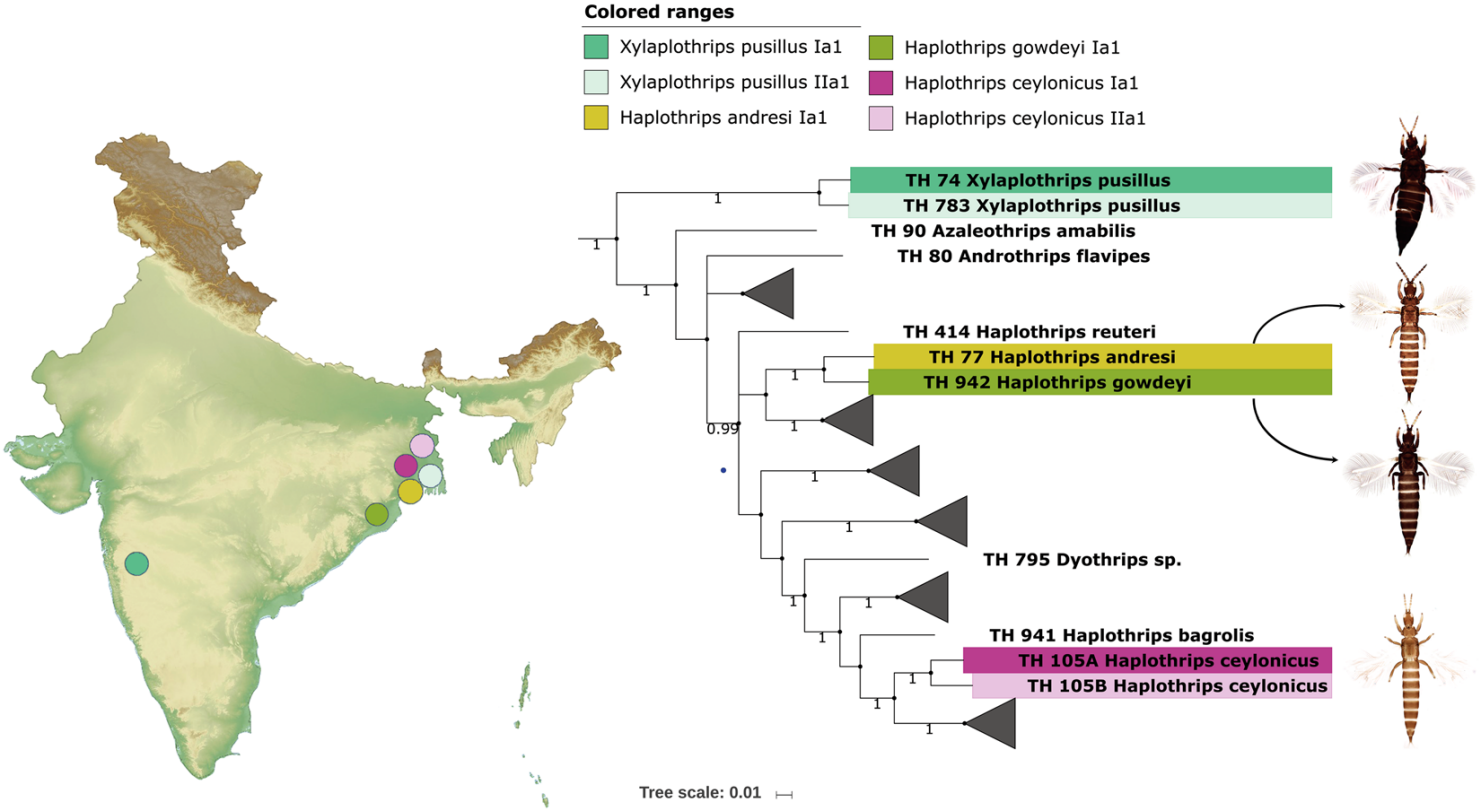
Pruned BA tree showing cryptic diversity of *Xylaplothrips pusillus* and *Haplothrips ceylonicus* with MOTUs nomenclature and geographical locations. BA tree also depicts low genetic distance between *Haplothrips andresi* and *Haplothrips gowdeyi*. Photographs used here were taken by the first author (K. T.). The original template of the topographic map used here is copied under the following attribution: created by Yug (Own work) [CC BY-SA 3.0 (http://creativecommons.org/licenses/by-sa/3.0)], via Wikimedia Commons; file URL: https://upload.wikimedia.org/wikipedia/commons/b/b9/Wikimaps_atlas-India-topographic_map-color-blank.jpg; page URL: https://commons.wikimedia.org/wiki/File%3AWikimaps_atlas-India-topographic_map-color-blank.jpg.

The cryptic speciation in *A*. *distinctus*, *F*. *megalops*, *M*. *nilgiriensis*, and *T*. *alatus* was detected with low sample size. These four species need additional specimens to make further assumptions about the validity of cryptic species and are thus not discussed further in this paper. Out of 12 cryptic species, six morphospecies (*A*. *intermedius*, *S. oligochaetus*, *T*. *apicatus*, *T*. *andrewsi*, *H*. *ceylonicus*, *X*. *pusillus*) may be considered in future studies coupled with more data (Figs 3b, 5c, 6).

### Distinct morphospecies with low genetic distance

Two morphospecies, *Thrips moundi* and *T. carthami* are represented by six and seven specimens respectively. The NJ tree showed two clades for these two species with a low bootstrap support value. The BA tree detected the 12 specimens of two species in one clade and one specimen of *T. moundi* in second clade. The K2P intraspecific distances in *T. moundi* (0.0071 to 0.0107) and *T. carthami* (0.0035 to 0.0179) overlap with very low interspecific distance (0.0135). The patristic intraspecific distances *T. moundi* (0.0092 to 0.0128) and *T. carthami* (0.0062 to 0.0218) also overlap with minimum interspecific distance (0.0166 to 0.0340). The four automatic delimitation methods provide different results. The data from ABGD and bPTP considered these two species in a single MOTU. Whereas, GMYC treated 12 specimens in one MOTU and one specimen (TH-641) of *T. moundi* in a second MOTU. The BIN analysis results were similar to the morphological identification as it divides these two species into three MOTUs; one MOTU for *T. moundi* specimens and two MOTUs in *T. carthami* specimens. We detected two MOTUs each in *T. moundi* (*T. moundi* Ia1 and *T. moundi* Ia2) and *T. carthami* (*T. carthami* Ia1 and *T. carthami* Ia2) by superimposing all the four species delimitation methods (Fig. 5a). Further, there is no haplotype sharing between these two morphospecies.

## Discussion

In this study, we examined the utility of DNA barcode data in species identification and existence of cryptic species in 89 morphospecies of thrips. Using multiple analysis methods for DNA barcode data, our study indicated cryptic speciation in six morphospecies (*A. distinctus*, *F. schultzei*, *F. megalops*, *M. nilgiriensis*, *T. alatus* and *T. palmi*). The results suggest that in these cryptic species, separation happened through high substitution rate regardless of change in morphological features. Contrary to this, we found that four valid distinguishable morphospecies (*T. carthami*; *T. moundi, and H*. *andresi*; *H*. *gowdeyi*) have a low genetic distance.

In our study, the mean K2P intraspecific genetic distance was found to be 0.0101 (0.00 to 0.1854) and mean K2P interspecific genetic distance was found to be 0.1871 (0.0135 to 0.2775). The higher mean intraspecific genetic distance is due to the presence of cryptic diversity in *F. schultzei* and *T. palmi* where maximum intraspecific genetic distance was recorded 0.1854 and 0.0829 respectively. Excluding these two species, the overall mean K2P intraspecific distance drops down to 0.0073 and compared to 0.1871 interspecific mean genetic distance. The same pattern of a distinct gap in intra and interspecific distance was observed in patristic distances.

### Resolving species complex

Barcode data analysis successfully distinguished between closely related species and species complexes. *Thrips alatus*, known as ‘Himalayan *palmi*’^30^, can be easily misidentified as *T. palmi*. However, the distances (K2P and patristic), NJ and BA tree analysis and results of four automated delimitation methods clearly distinguish these two species. Our study also resolved historical ‘*hawaiiensis* species complex’ containing three morphospecies; *T. hawaiiensis*, *T. andrewsi*, and *T. florum*. We analyzed multiple samples of both sexes for each of these species; *T. hawaiiensis* (10), *T. florum* (8), and *T. andrewsi* (10). These three species are difficult to separate morphologically from each other, as specimens are often collected together and display sexual dimorphism in body color, females being dark brown to bicolored while the males are completely pale yellow^56^. Further, *T. hawaiiensis* and *T. florum* were synonymized under each other^57^. The thrips species *T. hawaiiensis* is a global pest on a wide variety of plants and look-alike *T. florum*. It is suggested that some of the damage caused by *T. hawaiiensis* may be attributed to *T. florum* ^58^.

### Cryptic species

Recent barcoding studies on thrips have revealed cryptic speciation in a number of pest and vector species, eg. *F. occidentalis* ^7^, *T. palmi* ^27, 28^, *F. schultzei* ^59^. However, the cryptic species in earlier studies were from widely separated geographical areas; which probably shows allopatric speciation. Further, the inconspicuous morphology and lack of voucher specimens restrict the reliability of these cryptic species identification in the genus *Frankliniella* and *Thrips*. Therefore, it is to be ascertained whether i) these cryptic species are new to science ii) closely related species tagged with the incorrect name and iii) a synonym that needs to be resurrected. Thus, detection of cryptic species in any economically important taxon should be considered with extreme care for pest and vector management.

In the current study, two important pest and vector species, *F. schultzei* and *T. palmi*, were detected as involving cryptic species. Both the species have worldwide distribution, coupled with serious economic losses as direct crop pests and as vectors of Tospoviruses, and have identification problems that are reflected in their long list of synonyms^1, 30^. *F. schultzei*, which is reported as a vector for at least five tospovirses has been suspected to be a complex of more than one species^20, 59^. It is one of the historical unresolved issues in thrips taxonomy where, two species namely, *F. schultzei* Trybom, 1910 and *F. sulphurea* Schmutz, 1913 were erected from South Africa and Sri Lanka respectively. These two species were considered to be valid until 1968, when *sulphurea* was treated as color morph (pale form) and synonymized under *schultzei* (dark form), as there were no distinguishing morphological characters and the characters used to distinguish these two species were found to be variable within populations^60^. However, these two have been considered as a valid species by some of thrips workers^61^. Parallel studies have indicated that both the brown and yellow form of *schultzei* are vector for tospoviruses^1, 62^. In this study, we recovered three cryptic species in *F. schultzei*, from three different geographical regions: *F. schultzei* Ia1, *F. schultzei* IIa1, and *F. schultzei* IIIa1. Specimens of *F. schultzei* Ia1 were collected from the Rajasthan state in western India and are brown in color; *F. schultzei* IIa1 were collected from the Odisha state in eastern India and are completely pale yellow without a tinge of brown; *F. schultzei* IIIa1 were collected from the Karnataka state in southern India were completely dark brown in color. The color of *F. schultzei* IIa1 was similar to *F. sulphurea* while the color of *F. schultzei* Ia1 and *F. schultzei* IIIa1 was similar to *F. schultzei*. Support for these three cryptic species was provided by all the methods used in the study. These results indicate the possibility that *F. sulphurea* may be a valid species, however, it needs more morphological and molecular data on specimens collected from type locations of these two species and comparison of voucher specimens with type specimens. Further, for *F. schultzei* Ia1 and *F. schultzei* IIIa1, it is not certain which of these cryptic species would represent *schultzei* and which one is to be tested for some other hypothesis.

The morphospecies *Thrips palmi* was indicated with three cryptic species, *T. palmi* Ia1, *T. palmi* IIa1, and *T. palmi* Ib1+Ib2 corresponding to four MOTUs. Specimens of *T*. *palmi* Ia1 were collected from Odisha and West Bengal states in eastern India, Rajasthan state in western India and Himachal Pradesh state in northern India; *T. palmi* IIa1 and *T*. palmi Ib1 were collected from Odisha state in eastern India; *T. palmi* Ib2 was from Karnataka state in southern India. High genetic distances between these MOTUs (0.058 to 0.1004) indicate the possibility of cryptic species. Although we could not observe any morphological difference between these specimens, the three cryptic species show allopatric speciation in India. However, the presence of *T. palmi* Ia1, *T. palmi* IIa1, and *T. palmi* Ib1 in the same geographical location in Odisha state, possibly indicates sympatric speciation. This finding is important in the light of the fact that watermelon bud necrosis tospovirus caused by tospoviruses is vectored by *T. palmi* and is prevalent in India^63^. Thus, the presence of these cryptic species in DNA barcode sequences for *T. palmi* warrants serious future taxonomic studies coupled with data from multiple molecular markers and virus vector relationships.

### Incongruence between morphology and DNA barcoding

One of the advantages of using multiple delimitation methods was observed in identifying two valid morphospecies though DNA barcode data. *Thrips moundi* is a recently described species^33^ and member of ‘*Thrips formosanus* species group’, and it is closely related to *T. tanicus*, *T. floreus*, *T. formosanus*, *T. obscuripes*, and *T. rostratus*. It can be distinguished easily from *T. carthami* by the body color and chaetotaxy. However, the DNA barcode results are incongruent with the morphological identification of these two morphospecies by tree building methods and three automatic delimitation methods (ABGD, GMYC, and bPTP) except BIN. This incongruence may be due to incomplete lineage sorting of ancestral mitochondrial DNA polymorphisms, introgression of mitochondrial DNA, or due to Wolbachia infection causing genetic variability^64^. Additional data on morphology with multiple molecular markers on more specimens are required to resolve the inconsistency between the morphology and DNA barcode data. Similarly, a low genetic distance observed between two valid morphospecies of the genus *Haplothrips*; *H*. *andrewsi, and H*. *gowdeyi*. These two species can be distinguished by morphological characters, eg. number of sense cones on antennal segment III, and shape of the post ocular setae on head. However, three out of four delimitation methods distinguished these two morphospecies except GMYC.

The discrepancies in the results were observed when two MOTUs were detected in one morphospecies only by one of the species delimitation methods. These inconsistencies will be useful in future when more data becomes available for those morphospecies. Further, a three level nomenclature system applied here for the cryptic species will provide a strong framework for researchers to identify individuals according to MOTUs. It will provide a basis for morphological taxonomy to assess the validity of these MOTUs in future.

DNA barcoding since its inception has shown potential in for accurate and rapid identification of species. Large scale barcode data for economically important taxa like Thysanoptera can provide a common platform to researchers from wide array biological studies such as taxonomy, ecology, behavior, life histories, pest management, vector-virus relationship etc. Besides routine identification, DNA barcode data also provide insights into further taxonomic research through elucidation of cryptic species, resolving species complexes, new species descriptions etc. However, it is of prime importance that the name tagged with the generated sequences must be of high accuracy to utilize DNA barcode data for routine identification by other biologists. A significant number of DNA barcode sequences, either with wrong names or without taxonomic names, are frequently encountered in a wide variety of taxa^65^. Hence, the development of large scale barcode data for any taxa in the absence of a trained taxonomist may be misleading rather than helpful. Incorrect sequence tagging may not only alter the idea of accurate identification but also provide wrong signals for the crossroad of speciation and other serious taxonomic studies. The current study provides large scale DNA barcoding data for the identification of thrips species. This study contributed 104 novel sequences of 39 morphospecies to GenBank database, including types of four species identified by a trained taxonomist (first author). Most of the species included in this study are serious pests on a wide variety of agricultural and horticultural crops and are routinely collected by economic entomologists involved in various biological studies^4–6^.

## Electronic supplementary material

Supplementary Info
