# Supplementary material for: DNA Barcoding studies on Thrips in India: Cryptic species and Species complexes
Source: Sci Rep. 2017 Jul 7;7:4898. doi: 10.1038/s41598-017-05112-7 (PMC5501822; doi:10.1038/s41598-017-05112-7)
Supplement: Supplementary file 1 — Supplementary Info [file 41598_2017_5112_MOESM1_ESM.pdf]

DNA Barcoding studies on Thrips in India: Cryptic species and Species complexes

Kaomud Tyagi, Vikas Kumar*, Devkant Singha, Kailash Chandra, Boni Amin Laskar, Shantanu Kundu, Rajasree Chakraborty, Sumantika Chatterjee

Centre for DNA Taxonomy, Molecular Systematics Division, Zoological Survey of India, M- Block, New Alipore, Kolkata- 700 053, West Bengal, India

**Corresponding author's Email: vikaszsi77@gmail.com*

Figure S1: Sampling sites of the studied thrips species in India. Map not to scale. The original template of the topographic map used here is copied under the following attribution: created by Yug (Own work) [CC BY-SA 3.0 (http://creativecommons.org/licenses/by-sa/3.0)], via Wikimedia Commons; file URL: https://upload.wikimedia.org/wikipedia/commons/b/b9/Wikimaps_atlas-India-topographic_map-color-blank.jpg; page URL: https://commons.wikimedia.org/wiki/File%3AWikimaps_atlas-India-topographic_map-color-blank.jpg. The world map inset used here is copied under the following attribution: created by Addicted04 (Own work) [CC BY-SA 3.0 (http://creativecommons.org/licenses/by-sa/3.0)], via Wikimedia Commons; file URL: https://upload.wikimedia.org/wikipedia/commons/7/72/India_on_the_globe_%28India_centered%29.svg; page URL: https://commons.wikimedia.org/wiki/File%3AIndia_on_the_globe (India_centered).svg. The map was edited manually in Adobe Photoshop CS 8.0. to provide the details of sampling.

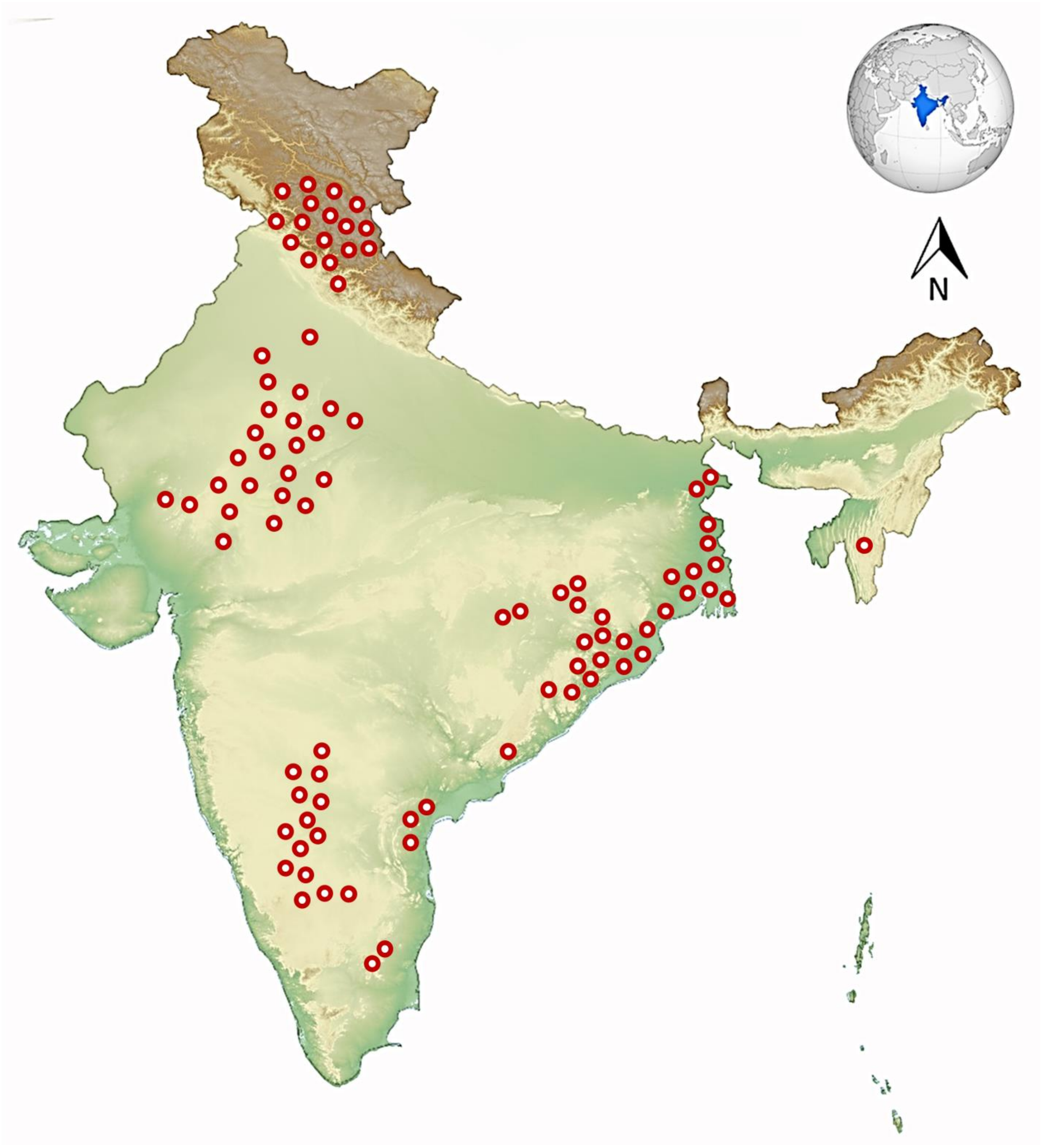

Figure S2. **Photographic Illustration of the studied voucher specimens.** Name of the species is given against the Arabic numerical. The photographs were taken by the first author (K. T.). Species names are: (1) *Aduncothrips asiaticus*; (2) *Aeolothrips distinctus*; (3) *A. intermedius*; (4) *Franklinothrips megalops*; (5) *F. vespiformis*; (6) *Mymarothrips garuda*; (7) *Streothrips* sp., male; (8) *Holarthrothrips indicus*; (9) *Astrothrips stannardi*; (10) *A. tumiceps*; (11) *Caliothrips indicus*; (12) *Heliothrips haemorrhoidalis*; (13) *Helionothrips aino*; (14) *H. parvus*; (15) *Phibalothrips peringueyi*; (16) *Retithrips syriacus*; (17) *Rhipiphorothrips cruentatus*; (18) *Selenothrips rubrocinctus*; (19) *Neohydatothrips chandrai*; (20) *N. plumeria*; (21) *N. samayunkur*; (22) *Anaphothrips obscurus*; (23) *A. sudanensis*; (24) *Aptinothrips rufus;* (25) *Ayyaria chaetophora*; (26) *Bathrips melanicornis*; (27) *Biltothrips minutus*; (28) *Bolacothrips striatopennatus*; (29) *Chirothrips africanus*; (30) *Dendrothripoides innoxius*; (31) *Diarthrothrips nimbus*; (32) *Eremiothrips antelope*; (33) *Exothrips ornus*; (34) *Florithrips traegardhi*; (35) *Frankliniella intonsa*; (36) *F. schultzei*; (37) *F. unicolor*; (38) *Lefroyothrips lefroyi*; (39) *Megalurothrips typicus*; (40) *Microcephalothrips abdominalis*; (41) *Moundinothrips robustus*; (42) *Mycterothrips nilgiriensis*; (43) *Oxythrips kochummani*; (44) *Parabaliothrips takahashii*; (45) *Scirtothrips dorsalis*; (46) *S. kenyensis*; (47) *S. oligochaetus*; (48) *Scirtothrips* sp.; (49) *Smilothrips productus*; (50) *Stenchaetothrips biformis*; (51) *S. pteratus*; (52) *S. spinulae*; (53) *Taeniothrips bharokariiensis*; (54) *Tenothrips frici*; (55) *Thrips alatus*; (56) *T. andrewsi*; (57) *T. apicatus*; (58) *T. carthami*; (59) *T. coloratus*; (60) *T. flavus*; (61) *T. florum*; (62) *T. hawaiiensis*; (63) *T. moundi*; (64) *T. orientalis*; (65) *T. palmi*; (66) *T. parvispinus*; (67) *T. subnudula*; (68) *T. tabaci*; (69) *Tusothrips sumatrensis*; (70) *Androthrips flavitibia*; (71) *Arrhenothrips acuminatus*; (72) *Azaleothrips amabilis*; (73) *Dolichothrips ochripes*; (74) *D. citripes*; (75) *Dyothrips* sp.; (76) *Gynaikothrips uzeli*; (77) *Plicothrips apicalis*; (78) *Haplothrips andrewsi*; (79) *H. bagrolis*; (80) *H. ceylonicus* (81) *H. ganglbaueri*; (82) *H. gowdeyi*; (83) *H. reuteri*; (84) *H. tenuipennis*; (85) *Xylaplothrips pusillus*; (86) *Idiothrips ficus* (87) Phaleothripid.

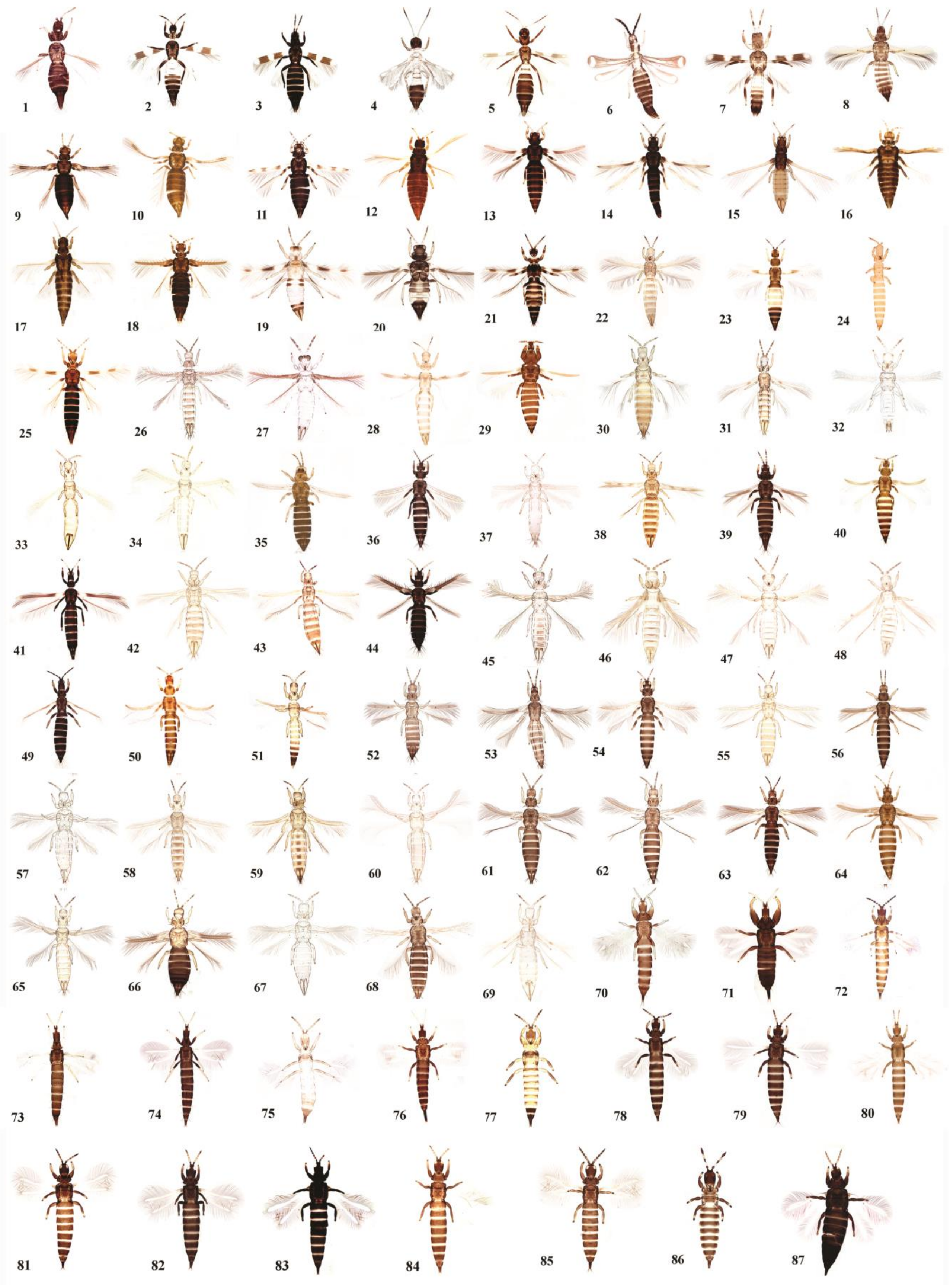
1 2 3 4 5 6 7 8
9 10 11 12 13 14 15 16
17 18 19 20 21 22 23 24
25 26 27 28 29 30 31 32
33 34 35 36 37 38 39 40
41 42 43 44 45 46 47 48
49 50 51 52 53 54 55 56
57 58 59 60 61 62 63 64
65 66 67 68 69 70 71 72
73 74 75 76 77 78 79 80
81 82 83 84 85 86 87

Figure S3: Bayesian inference gene tree with 138 unique sequences of the studied 89 thrips species.

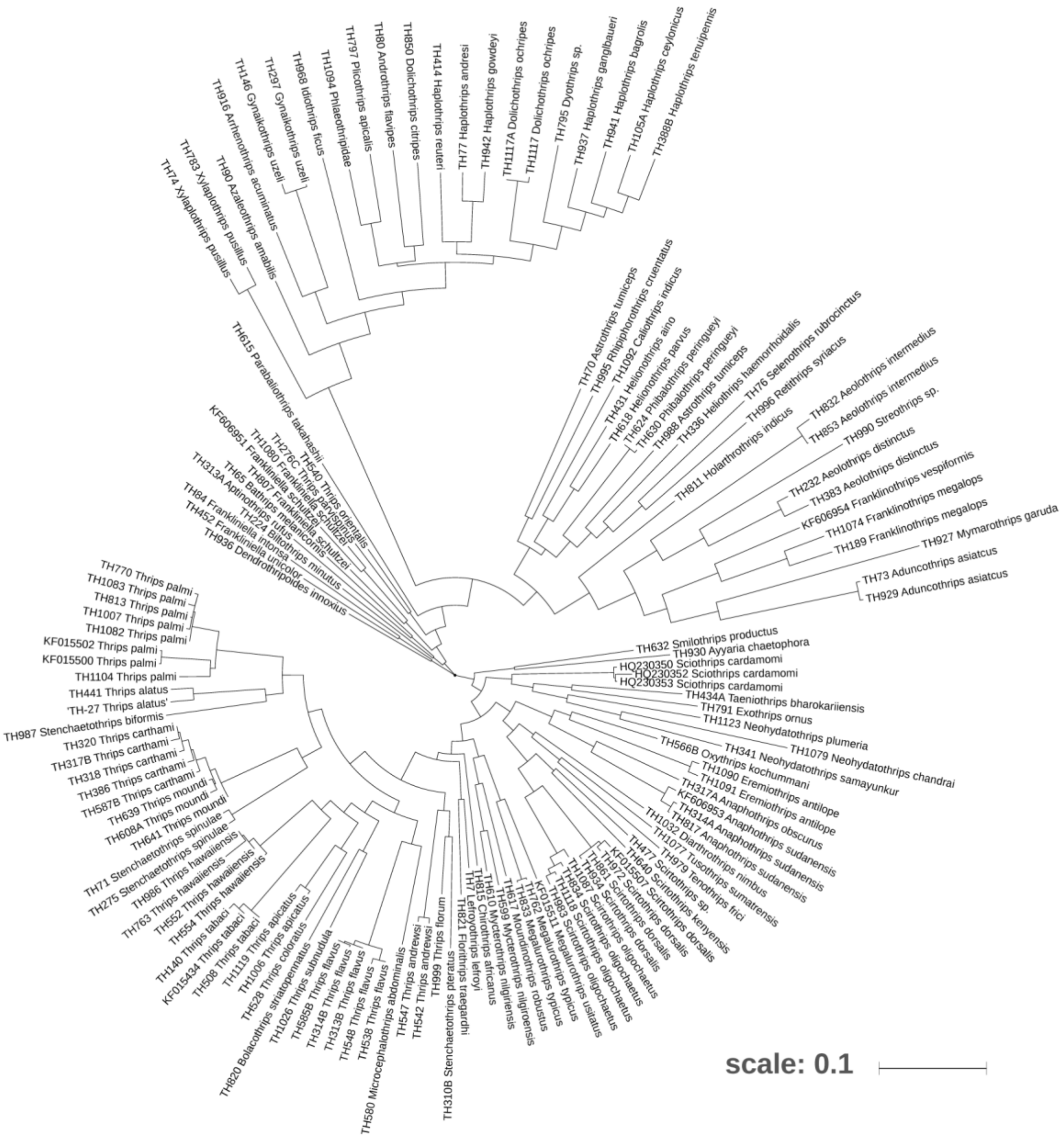

Figure S4: Neighbor-Joining tree of the studied 89 Thysanoptera species with 1000 bootstrap support.

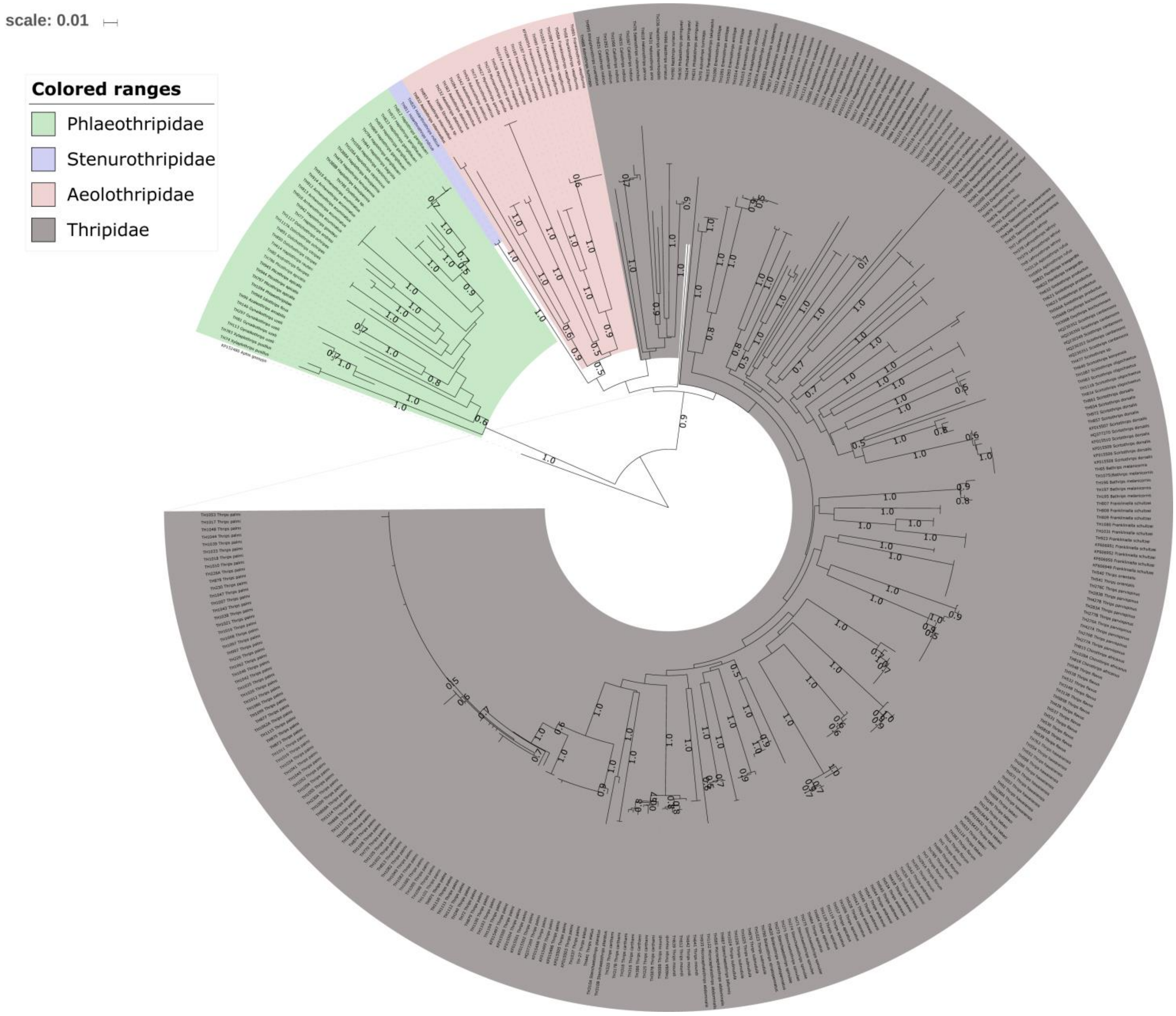

Table S1: Sample ID, Voucher ID, Species Name, BOLD Process IDs, GenBank Accession numbers, Collection date and locality and estimated haplotypes for each specimen.

Sl No.	Sample ID	Voucher ID	Species	BOLD process ID	Gen Bank Acc. No.	Collection Date	Lat	Long	Haplotype
1	TH-929	ZSI_CDT_TH-929	*Aduncothrips asiatcus*	BOLD:ACI5305	KX622170	8-Jun-15	11.6646	78.146	H12
2	TH-73	ZSI_CDT_TH-73	*Aduncothrips asiatcus*	BOLD:ACI5305	KF840078	14-Feb-13	12.7687	75.2071	H13
3	TH-383	ZSI_CDT_TH-383	*Aeolothrips distinctus*	BOLD:ACY8456	KX622171	15-Oct-13	32.139	76.4534	H14
4	TH-384	ZSI_CDT_TH-384	*Aeolothrips distinctus*	BOLD:ACY8456	KX622172	15-Oct-13	31.973	76.8488	H14
5	TH-342	ZSI_CDT_TH-342	*Aeolothrips distinctus*	BOLD:ACY8456	KX622173	14-Oct-13	32.2176	76.3061	H14
6	TH-232	ZSI_CDT_TH-232	*Aeolothrips distinctus*	BOLD:AAN6626	KX622174	12-Oct-13	32.5468	76.0562	H17
7	TH-832	ZSI_CDT_TH-832	*Aeolothrips intermedius*	BOLD:AAZ8618	KX622175	21-Dec-14	26.7509	75.9517	H15
8	TH-853	ZSI_CDT_TH-853	*Aeolothrips intermedius*	BOLD:AAZ8618	KX622176	22-Dec-14	26.6849	75.9002	H16
9	TH-189	ZSI_CDT_TH-189	*Franklinothrips megalops*	BOLD:ACM2095	KJ658420	25-Sep-13	22.5667	88.3667	H45
10	TH-185	ZSI_CDT_TH-185	*Franklinothrips megalops*	BOLD:ACM2095	KJ658418	25-Sep-13	22.5667	88.3667	H45
11	TH-187	ZSI_CDT_TH-187	*Franklinothrips megalops*	BOLD:ACM2095	KJ658419	25-Sep-13	22.5667	88.3667	H45
12	TH-1074	ZSI_CDT_TH-1074	*Franklinothrips megalops*		KX622224	31-May-15	20.2704	85.8063	H46
13	TH-989	ZSI_CDT_TH-989	*Franklinothrips vespiformis*	BOLD:ACD8336	KX622225	29-Dec-14	24.6727	73.8775	H1
14	TH-1003	ZSI_CDT_TH-1003	*Franklinothrips vespiformis*	BOLD:ACD8336	KX622226	29-Dec-14	24.6727	73.8775	H1
15	TH-1088	ZSI_CDT_TH-1088	*Franklinothrips vespiformis*	BOLD:ACD8336	KX622227	28-Dec-14	24.5686	73.818	H1
16	TH-58A	ZSI_CDT_TH-58A	*Franklinothrips vespiformis*	BOLD:ACD8336	KF840083	2-Feb-13	21.2787	81.8661	H1
17	TH-58	ZSI_CDT_TH-58	*Franklinothrips vespiformis*	BOLD:ACD8336	KF840084	2-Feb-13	21.2787	81.8661	H1
18	TH-991	ZSI_CDT_TH-991	*Franklinothrips vespiformis*	BOLD:ACD8336	KX622228	8-Jun-15	11.6646	78.146	H1
19	TH-927	ZSI_CDT_TH-927	*Mymarothrips garuda*	BOLD:ACL6651	KX622263	8-Jun-15	11.6643	78.146	H70
20	TH-270	ZSI_CDT_TH-270	*Mymarothrips garuda*	BOLD:ACL6651	KF577749	13-Jan-14	22.5602	88.4421	H70
21	TH-928	ZSI_CDT_TH-928	*Mymarothrips garuda*	BOLD:ACL6651	KX622264	8-Jun-15	11.6646	78.146	H70
22	TH-990	ZSI_CDT_TH-990	*Streothrips sp.*	BOLD:ACY9996	KX622306	29-Dec-14	24.6727	73.8775	H97
23	TH-811	ZSI_CDT_TH-811	*Holarthrothrips indicus*	BOLD:ACY8375	KX622250	30-Dec-14	26.7509	75.9517	H61
24	TH-825	ZSI_CDT_TH-825	*Holarthrothrips indicus*	BOLD:ACY8375	KX622251	21-Dec-14	26.7509	75.9517	H61
25	TH-70	ZSI_CDT_TH-70	*Astrothrips stannardi*	BOLD:ACI7059	KF840079	23-Feb-13	22.51	88.334	H24
26	TH-988	ZSI_CDT_TH-988	*Astrothrips tumiceps*	BOLD:ACY9954	KX622195	29-Dec-14	24.6727	73.8775	H25
27	TH-1092	ZSI_CDT_TH-1092	*Caliothrips indicus*	BOLD:ACY7911	KX622201	2-Jan-15	26.2356	72.9857	H31
28	TH-1066	ZSI_CDT_TH-1066	*Caliothrips indicus*	BOLD:ACY7911	KX622202	2-Jan-15	26.2356	72.9857	H31
29	TH-931	ZSI_CDT_TH-931	*Caliothrips indicus*	BOLD:ACY7911	KX622203	2-Jun-15	20.2704	85.8063	H31
30	TH-1067	ZSI_CDT_TH-1067	*Caliothrips indicus*	BOLD:ACY7911	KX622204	2-Jan-15	26.2356	72.9857	H31
31	TH-831	ZSI_CDT_TH-831	*Caliothrips indicus*	BOLD:ACY7911	KX622205	21-Dec-14	26.7509	75.9517	H31
32	TH-618	ZSI_CDT_TH-618	*Helionothrips parvus*	BOLD:ACY8245	KX622247	12-Jun-14	31.0583	77.2719	H58
33	TH-431	ZSI_CDT_TH-431	*Helionothrips aino*	BOLD:ACY8267	KX622248	4-Jun-14	31.427	77.0746	H59
34	TH-336	ZSI_CDT_TH-336	*Heliothrips haemorrhoidalis*	BOLD:AAE5223	KX622249	13-Oct-13	32.2023	76.3147	H60
35	TH-624	ZSI_CDT_TH-624	*Phibalothrips peringueyi*	BOLD:ACY8031	KX622275	20-Jul-14	27.05	88.2667	H76
36	TH-631	ZSI_CDT_TH-631	*Phibalothrips peringueyi*	BOLD:ACY8031	KX622276	8-Jun-14	31.7724	77.2	H76
37	TH-630	ZSI_CDT_TH-630	*Phibalothrips peringueyi*	BOLD:ACY8031	KX622277	8-Jun-14	31.7724	77.2	H77
38	TH-996	ZSI_CDT_TH-996	*Retithrips syriacus*	BOLD:ACY7969	KX622283	2-Jun-15	20.2857	85.7916	H80

39	TH-780	ZSI_CDT_TH-780	*Retithrips syriacus*	BOLD:ACY7969	KX622284	7-Dec-14	22.5667	88.3667	H80
40	TH-995	ZSI_CDT_TH-995	*Rhipiphorothrips cruentatus*	BOLD:ACY9953	KX622285	2-Jun-15	20.2857	85.7916	H81
41	TH-76	ZSI_CDT_TH-76	*Selenothrips rubrocinctus*	BOLD:ACN6317	KF840091	14-Feb-13	12.77	75.22	H91
42	TH-1079	ZSI_CDT_TH-1079	*Neohydatothrips chandrai*	BOLD:ACG8261	KX622270	1-Jun-15	20.2704	85.8063	H72
43	TH-1123	ZSI_CDT_TH-1123	*Neohydatothrips plumeria*	BOLD:ACZ0079	KX622271	22-Apr-14	22.5667	88.3667	H73
44	TH-341	ZSI_CDT_TH-341	*Neohydatothrips samayunkur*	BOLD:AAP7680	KX622265	14-Oct-13	32.2177	76.3061	H71
45	TH-939	ZSI_CDT_TH-939	*Neohydatothrips samayunkur*	BOLD:AAP7680	KX622266	28-Dec-14	24.5691	73.7005	H71
46	TH-1001	ZSI_CDT_TH-1001	*Neohydatothrips samayunkur*	BOLD:AAP7680	KX622267	29-Dec-14	24.885	74.3338	H71
47	TH-309	ZSI_CDT_TH-309	*Neohydatothrips samayunkur*	BOLD:AAP7680	KX622268	11-Oct-13	32.5507	76.0188	H71
48	TH-1000	ZSI_CDT_TH-1000	*Neohydatothrips samayunkur*	BOLD:AAP7680	KX622269	29-Dec-14	24.885	74.3338	H71
49	TH-317A	ZSI_CDT_TH-317A	*Anaphothrips obscurus*	BOLD:AAQ0558	KX622177	12-Oct-13	32.5468	76.0562	H18
50	TH-587A	ZSI_CDT_TH-587A	*Anaphothrips obscurus*	BOLD:AAQ0558	KX622178	7-Jun-14	32.0993	77.136	H18
51	TH-314A	ZSI_CDT_TH-314A	*Anaphothrips sudanensis*	BOLD:AAV3388	KX622179	11-Oct-13	32.5506	76.0184	H19
52	TH-1121	ZSI_CDT_TH-1121	*Anaphothrips sudanensis*	BOLD:AAV3388	KX622180	26-Dec-14	26.3669	74.5849	H19
53	TH-1073	ZSI_CDT_TH-1073	*Anaphothrips sudanensis*	BOLD:AAV3388	KX622181	31-May-15	20.2704	85.8063	H19
54	TH-817	ZSI_CDT_TH-817	*Anaphothrips sudanensis*	BOLD:AAV3388	KX622182	21-Dec-14	26.7685	75.917	H20
55	TH-586	ZSI_CDT_TH-586	*Anaphothrips sudanensis*	BOLD:AAV3388	KX622183	7-Jun-14	32.1749	77.1795	H19
56	TH-581A	ZSI_CDT_TH-581A	*Anaphothrips sudanensis*	BOLD:AAV3388	KX622184	7-Jun-14	32.1749	77.1795	H19
57	TH-816	ZSI_CDT_TH-816	*Anaphothrips sudanensis*	BOLD:AAV3388	KX622185	21-Dec-14	26.7685	75.917	H19
58	TH-312	ZSI_CDT_TH-312	*Anaphothrips sudanensis*	BOLD:AAV3388	KX622186	11-Oct-13	32.5506	76.0184	H19
59	TH-313A	ZSI_CDT_TH-313A	*Aptinothrips rufus*	BOLD:ACY7882	KX622188	11-Oct-13	32.5506	76.0184	H22
60	TH-585A	ZSI_CDT_TH-585A	*Aptinothrips rufus*	BOLD:ACY7882	KX622189	7-Jun-14	32.1749	77.1795	H22
61	TH-930	ZSI_CDT_TH-930	*Ayyaria chaetophora*	BOLD:ACZ0116	KX622196	2-Jun-15	20.2704	85.8063	H26
62	TH-65	ZSI_CDT_TH-65	*Bathrips melanicornis*	BOLD:ACI6059	KF840092	23-Feb-13	19.2647	84.862	H28
63	TH-1075	ZSI_CDT_TH-1075	*Bathrips melanicornis*	BOLD:ACI6059	KX622198	31-May-15	20.2704	85.8063	H28
64	TH-197	ZSI_CDT_TH-197	*Bathrips melanicornis*	BOLD:ACI6059	KJ740613	12-Oct-13	32.5339	76.0275	H28
65	TH-195	ZSI_CDT_TH-195	*Bathrips melanicornis*	BOLD:ACI6059	KJ740611	10-Oct-13	32.5339	76.0275	H28
66	TH-196	ZSI_CDT_TH-196	*Bathrips melanicornis*	BOLD:ACI6059	KJ740612	11-Oct-13	32.5339	76.0275	H28
67	TH-224	ZSI_CDT_TH-224	*Biltothrips minutus*	BOLD:ACL7167	KF577746	13-Jan-14	22.5279	88.3625	H29
68	TH-269	ZSI_CDT_TH-269	*Biltothrips minutus*	BOLD:ACL7167	KF577748	13-Jan-14	22.5279	88.3625	H29
69	TH-268	ZSI_CDT_TH-268	*Biltothrips minutus*	BOLD:ACL7167	KF577747	13-Jan-14	22.5279	88.3625	H29
70	TH-223	ZSI_CDT_TH-223	*Biltothrips minutus*	BOLD:ACL7167	KF577745	13-Jan-14	22.5279	88.3625	H29
71	TH-820	ZSI_CDT_TH-820	*Bolacothrips striatopennatus*	BOLD:ACY8904	KX622199	21-Dec-14	26.7685	75.917	H30
72	TH-790	ZSI_CDT_TH-790	*Bolacothrips striatopennatus*	BOLD:ACY8904	KX622200	21-Dec-14	26.7512	75.9357	H30
73	TH-815	ZSI_CDT_TH-815	*Chirothrips africanus*	BOLD:ACR6888	KX622206	21-Dec-14	26.7685	75.917	H32
74	TH-1028A	ZSI_CDT_TH-1028A	*Chirothrips africanus*	BOLD:ACR6888	KX622207	26-Dec-14	26.3669	74.5849	H32
75	TH-818	ZSI_CDT_TH-818	*Chirothrips africanus*	BOLD:ACR6888	KX622208	21-Dec-14	26.7685	75.917	H32
76	TH-1032	ZSI_CDT_TH-1032	*Diarthrothrips nimbus*	BOLD:ACP4916	KX622210	2-Jun-15	20.2857	85.7916	H34
77	TH-936	ZSI_CDT_TH-936	*Dendrothripoides innoxius*	BOLD:ACZ0271	KX622209	8-Jun-15	22.51	88.32	H33
78	TH-1091	ZSI_CDT_TH-1091	*Eremiothrips antilope*	BOLD:ACZ0015	KX622216	3-Jan-15	26.2356	72.9857	H39

79	TH-1093	ZSI_CDT_TH-1093	*Eremiothrips antilope*	BOLD:ACZ0015	KX622217	2-Jan-15	26.2356	72.9857	H39
80	TH-1014	ZSI_CDT_TH-1014	*Eremiothrips antilope*	BOLD:ACZ0015	KX622218	2-Jan-15	26.2356	72.9857	H39
81	TH-1015	ZSI_CDT_TH-1015	*Eremiothrips antilope*	BOLD:ACZ0015	KX622219	2-Jan-15	26.2356	72.9857	H39
82	TH-1090	ZSI_CDT_TH-1090	*Eremiothrips antilope*	BOLD:ACZ0015	KX622220	3-Jan-15	26.2356	72.9857	H40
83	TH-791	ZSI_CDT_TH-791	*Exothrips ornus*	BOLD:ACY8436	KX622221	21-Dec-14	26.7512	75.9357	H41
84	TH-821	ZSI_CDT_TH-821	*Florithrips traegardhi*	BOLD:AAN9111	KX622222	21-Dec-14	26.7685	75.917	H42
85	TH-822	ZSI_CDT_TH-822	*Florithrips traegardhi*	BOLD:AAN9111	KX622223	21-Dec-14	26.7685	75.917	H42
86	TH-84	ZSI_CDT_TH-84	*Frankliniella intonsa*	BOLD:AAF6737	KF840080	23-Feb-13	22.51	88.334	H43
87	TH-1080	ZSI_CDT_TH-1080	*Frankliniella schultzei*	BOLD:ACY9272	KX622449	1-Jun-15	20.2704	85.8063	H137
88	TH-1031	ZSI_CDT_TH-1031	*Frankliniella schultzei*	BOLD:ACY9272	KX622450	2-Jun-15	20.2857	85.7916	H137
89	TH-807	ZSI_CDT_TH-807	*Frankliniella schultzei*	BOLD:AAM8089	KX622451	3-Jun-15	24.7346	73.9191	H138
90	TH-808	ZSI_CDT_TH-808	*Frankliniella schultzei*	BOLD:AAM8089	KX622452	4-Jun-15	24.7346	73.9191	H138
91	TH-809	ZSI_CDT_TH-809	*Frankliniella schultzei*	BOLD:AAM8089	KX622453	29-Dec-14	24.7346	73.9191	H138
92	TH-923	ZSI_CDT_TH-923	*Frankliniella schultzei*	BOLD:ACY9272	KX622454	1-Jun-15	20.5121	85.97	H137
93	TH-452	ZSI_CDT_TH-452	*Frankliniella unicolor*	BOLD:ACR6111	KP278562	4-Jun-14	31.4175	77.09	H44
94	TH-451B	ZSI_CDT_TH-451B	*Frankliniella unicolor*	BOLD:ACR6111	KP278561	4-Jun-14	31.4175	77.09	H44
95	TH-451A	ZSI_CDT_TH-451A	*Frankliniella unicolor*	BOLD:ACR6111	KP278560	4-Jun-14	31.4175	77.09	H44
96	TH-7	ZSI_CDT_TH-7	*Lefroyothrips lefroyi*	BOLD:ACI6048	KF840089	22-Nov-11	31.1048	77.1734	H63
97	TH-378	ZSI_CDT_TH-378	*Lefroyothrips lefroyi*	BOLD:ACI6048	KX622253	15-Oct-13	32.139	76.4534	H63
98	TH-379	ZSI_CDT_TH-379	*Lefroyothrips lefroyi*	BOLD:ACI6048	KX622254	15-Oct-13	32.139	76.4534	H63
99	TH-8	ZSI_CDT_TH-8	*Lefroyothrips lefroyi*	BOLD:ACI6048	KF840088	22-Nov-11	31.1048	77.1734	H63
100	TH-762	ZSI_CDT_TH-762	*Megalurothrips typicus*	BOLD:ACY7764	KX622255	1-Nov-14	23.1645	92.9376	H64
101	TH-833	ZSI_CDT_TH-833	*Megalurothrips typicus*	BOLD:ACY7764	KX622256	21-Dec-14	26.9018	75.8668	H65
102	TH-580	ZSI_CDT_TH-580	*Microcephalothrips abdominalis*	BOLD:AAI0410	KX622257	7-Jun-14	32.0247	77.1298	H66
103	TH-1122	ZSI_CDT_TH-1122	*Microcephalothrips abdominalis*	BOLD:AAI0410	KX622258	26-Dec-14	26.3669	74.5849	H66
104	TH-935	ZSI_CDT_TH-935	*Microcephalothrips abdominalis*	BOLD:AAI0410	KX622259	2-Jun-15	20.2704	85.8063	H66
105	TH-617	ZSI_CDT_TH-617	*Moundinothrips robustus*	BOLD:ACR0282	KP120982	13-Jun-14	31.1199	77.2312	H67
106	TH-610	ZSI_CDT_TH-610	*Mycterothrips nilgiriensis*	BOLD:AAP7685	KX622260	12-Jun-14	31.0583	77.2719	H68
107	TH-14	ZSI_CDT_TH-14	*Mycterothrips nilgiriensis*	BOLD:AAP7685	KF840090	17-Nov-12	31.1048	77.1734	H68
108	TH-438	ZSI_CDT_TH-438	*Mycterothrips nilgiriensis*	BOLD:AAP7685	KX622261	4-Jun-14	31.42	77.0761	H68
109	TH-599	ZSI_CDT_TH-599	*Mycterothrips nilgiriensis*	BOLD:ACY8466	KX622262	12-Jun-14	31.0582	77.2718	H69
110	TH-566B	ZSI_CDT_TH-566B	*Oxythrips kochummani*	BOLD:ACY8447	KX622272	6-Jun-14	32.0093	77.3102	H74
111	TH-306B	ZSI_CDT_TH-306B	*Oxythrips kochummani*	BOLD:ACY8447	KX622273	11-Oct-13	32.5507	76.0188	H74
112	TH-615	ZSI_CDT_TH-615	*Parabaliothrips takahashii*	BOLD:ACY7659	KX622274	13-Jun-14	31.1199	77.2312	H75
113	TH-972	ZSI_CDT_TH-972	*Scirtothrips dorsalis*	BOLD:ACV7644	KX622286	1-Jun-15	20.2705	85.8064	H82
114	TH-861	ZSI_CDT_TH-861	*Scirtothrips dorsalis*	BOLD:ACV7644	KX622287	22-Dec-14	26.8351	75.7845	H83
115	TH-934	ZSI_CDT_TH-934	*Scirtothrips dorsalis*	BOLD:ACV7644	KX622288	2-Jun-15	20.2704	85.8063	H84
116	TH-857	ZSI_CDT_TH-857	*Scirtothrips dorsalis*	BOLD:ACV7644	KX622289	22-Dec-14	26.8351	75.7845	H82
117	TH-477	ZSI_CDT_TH-477	*Scirtothrips sp.*	BOLD:ACY7963	KX622290	5-Jun-14	31.4342	77.0718	H85
118	TH-983	ZSI_CDT_TH-983	*Scirtothrips oligochaetus*	BOLD:AAZ8518	KX622291	28-Dec-14	24.5686	73.818	H86
119	TH-1118	ZSI_CDT_TH-1118	*Scirtothrips oligochaetus*	BOLD:AAZ8518	KX622292	25-Dec-14	26.2833	74.7516	H87
120	TH-834	ZSI_CDT_TH-834	*Scirtothrips oligochaetus*	BOLD:AAZ8518	KX622293	21-Dec-14	26.9018	75.8668	H88

121	TH-1087	ZSI_CDT_TH-1087	*Scirtothrips oligochaetus*	BOLD:AAZ8518	KX622294	28-Dec-14	24.5686	73.818	H89
122	TH-640	ZSI_CDT_TH-640	*Scirtothrips kenyensis*	BOLD:ACY7741	KX622295	12-Jun-14	31.0522	77.2684	H90
123	TH-632	ZSI_CDT_TH-632	*Smilothrips productus*	BOLD:ACY7742	KX622296	8-Jun-14	31.7724	77.2	H92
124	TH-621	ZSI_CDT_TH-621	*Smilothrips productus*	BOLD:ACY7742	KX622297	13-Jun-14	31.1109	77.2504	H92
125	TH-623	ZSI_CDT_TH-623	*Smilothrips productus*	BOLD:ACY7742	KX622298	20-Jul-14	27.05	88.2667	H92
126	TH-566A	ZSI_CDT_TH-566A	*Smilothrips productus*	BOLD:ACY7742	KX622299	6-Jun-14	32.0093	77.3102	H92
127	TH-987	ZSI_CDT_TH-987	*Stenchaetothrips biformis*	BOLD:ACY9843	KX622300	29-Dec-14	24.6727	73.8775	H93
128	TH-310B	ZSI_CDT_TH-310B	*Stenchaetothrips pteratus*	BOLD:ACY8903	KX622301	11-Oct-13	32.5507	76.0188	H94
129	TH-310A	ZSI_CDT_TH-310A	*Stenchaetothrips pteratus*	BOLD:ACY8903	KX622302	11-Oct-13	32.5507	76.0188	H94
130	TH-71	ZSI_CDT_TH-71	*Stenchaetothrips spinulae*	BOLD:ACI5207	KF840093	1-Mar-13	22.5008	88.3681	H95
131	TH-271	ZSI_CDT_TH-271	*Stenchaetothrips spinulae*	BOLD:ACI5207	KF577750	13-Feb-14	22.51	88.334	H95
132	TH-274	ZSI_CDT_TH-274	*Stenchaetothrips spinulae*	BOLD:ACI5207	KX622303	12-Feb-14	22.51	88.334	H95
133	TH-273	ZSI_CDT_TH-273	*Stenchaetothrips spinulae*	BOLD:ACI5207	KX622304	13-Feb-14	22.51	88.334	H95
134	TH-275	ZSI_CDT_TH-275	*Stenchaetothrips spinulae*	BOLD:ACI5207	KX622305	13-Feb-14	22.51	88.334	H96
135	TH-434A	ZSI_CDT_TH-434A	*Taeniothrips bharokariiensis*	BOLD:ACP1595	KM485668	4-Jun-14	31.427	77.0746	H98
136	TH-434B	ZSI_CDT_TH-434B	*Taeniothrips bharokariiensis*	BOLD:ACP1595	KM485669	4-Jun-14	31.427	77.0746	H98
137	TH-435	ZSI_CDT_TH-435	*Taeniothrips bharokariiensis*	BOLD:ACP1595	KM485670	4-Jun-14	31.427	77.0746	H98
138	TH-979	ZSI_CDT_TH-979	*Tenothrips frici*	BOLD:ACY9167	KX622307	1-Jan-15	26.2171	73.0344	H99
139	TH-978	ZSI_CDT_TH-978	*Tenothrips frici*	BOLD:ACY9167	KX622308	1-Jan-15	26.2171	73.0344	H99
140	TH-441	ZSI_CDT_TH-441	*Thrips alatus*		KX622309	4-Jun-14	31.4175	77.09	H100
141	TH-327	ZSI_CDT_TH-327	*Thrips alatus*	BOLD:AAN6625	KX622310	13-Oct-13	32.4896	75.9327	H101
142	TH-542	ZSI_CDT_TH-542	*Thrips andrewsi*	BOLD:ACA3048	KX622311	4-Jun-14	31.427	77.0746	H102
143	TH-536	ZSI_CDT_TH-536	*Thrips andrewsi*	BOLD:ACA3048	KX622312	6-Jun-14	31.942	77.1747	H102
144	TH-535	ZSI_CDT_TH-535	*Thrips andrewsi*	BOLD:ACA3048	KX622313	6-Jun-14	31.942	77.1747	H102
145	TH-418	ZSI_CDT_TH-418	*Thrips andrewsi*	BOLD:ACA3048	KX622314	4-Jun-14	31.427	77.0746	H102
146	TH-547	ZSI_CDT_TH-547	*Thrips andrewsi*	BOLD:ACA3048	KX622315	4-Jun-14	31.427	77.0746	H103
147	TH-546	ZSI_CDT_TH-546	*Thrips andrewsi*	BOLD:ACA3048	KX622316	4-Jun-14	31.427	77.0746	H103
148	TH-545	ZSI_CDT_TH-545	*Thrips andrewsi*	BOLD:ACA3048	KX622317	4-Jun-14	31.427	77.0746	H103
149	TH-544	ZSI_CDT_TH-544	*Thrips andrewsi*	BOLD:ACA3048	KX622318	4-Jun-14	31.427	77.0746	H103
150	TH-543	ZSI_CDT_TH-543	*Thrips andrewsi*	BOLD:ACA3048	KX622319	4-Jun-14	31.427	77.0746	H103
151	TH-534	ZSI_CDT_TH-534	*Thrips andrewsi*	BOLD:ACA3048	KX622320	6-Jun-14	31.942	77.1747	H102
152	TH-1006	ZSI_CDT_TH-1006	*Thrips apicatus*	BOLD:ACY8630	KX622321	6-Apr-15	22.5602	88.4421	H104
153	TH-1119	ZSI_CDT_TH-1119	*Thrips apicatus*	BOLD:AAY6262	KX622322	25-Dec-14	26.2833	74.7516	H105
154	TH-557	ZSI_CDT_TH-557	*Thrips apicatus*	BOLD:ACY8630	KX622323	26-Dec-14	15.3173	75.7139	H104
155	TH-1120	ZSI_CDT_TH-1120	*Thrips apicatus*	BOLD:AAY6262	KX622324	26-Dec-14	26.3669	74.5849	H105
156	TH-904	ZSI_CDT_TH-904	*Thrips apicatus*	BOLD:AAY6262	KX622325	25-Dec-14	26.486	74.5847	H105
157	TH-984	ZSI_CDT_TH-984	*Thrips apicatus*	BOLD:AAY6262	KX622326	28-Dec-14	24.5686	73.818	H105
158	TH-386	ZSI_CDT_TH-386	*Thrips carthami*	BOLD:ACY8520	KX622327	15-Oct-13	31.9731	76.8489	H106
159	TH-587B	ZSI_CDT_TH-587B	*Thrips carthami*	BOLD:AAP7682	KX622328	7-Jun-14	32.1749	77.1795	H107
160	TH-317B	ZSI_CDT_TH-317B	*Thrips carthami*	BOLD:AAP7682	KX622329	12-Oct-13	32.5468	76.0562	H108
161	TH-325	ZSI_CDT_TH-325	*Thrips carthami*	BOLD:AAP7682	KX622330	12-Oct-13	32.5468	76.0562	H107
162	TH-318	ZSI_CDT_TH-318	*Thrips carthami*	BOLD:AAP7682	KX622331	12-Oct-13	32.5468	76.0562	H109

163	TH-316	ZSI_CDT_TH-316	*Thrips carthami*	BOLD:AAP7682	KX622332	12-Oct-13	32.5468	76.0562	H107
164	TH-320	ZSI_CDT_TH-320	*Thrips carthami*	BOLD:AAP7682	KX622333	12-Oct-13	32.5468	76.0562	H110
165	TH-528	ZSI_CDT_TH-528	*Thrips coloratus*	BOLD:AAK1804	KX622334	13-Oct-13	15.9129	79.74	H111
166	TH-548	ZSI_CDT_TH-548	*Thrips flavus*	BOLD:AAN6624	KX622335	4-Jun-14	31.427	77.0746	H112
167	TH-313B	ZSI_CDT_TH-313B	*Thrips flavus*	BOLD:AAN6624	KX622336	11-Oct-13	32.5507	76.0188	H113
168	TH-585B	ZSI_CDT_TH-585B	*Thrips flavus*	BOLD:AAN6624	KX622337	7-Jun-14	32.1749	77.1795	H114
169	TH-428	ZSI_CDT_TH-428	*Thrips flavus*	BOLD:AAN6624	KX622338	4-Jun-14	31.427	77.0746	H113
170	TH-532	ZSI_CDT_TH-532	*Thrips flavus*	BOLD:AAN6624	KX622339	6-Jun-14	31.942	77.1747	H113
171	TH-537	ZSI_CDT_TH-537	*Thrips flavus*	BOLD:AAN6624	KX622340	6-Jun-14	31.942	77.1747	H113
172	TH-531	ZSI_CDT_TH-531	*Thrips flavus*	BOLD:AAN6624	KX622341	6-Jun-14	31.942	77.1747	H113
173	TH-530	ZSI_CDT_TH-530	*Thrips flavus*	BOLD:AAN6624	KX622342	6-Jun-14	31.942	77.1747	H113
174	TH-581B	ZSI_CDT_TH-581B	*Thrips flavus*	BOLD:AAN6624	KX622343	7-Jun-14	32.1749	77.1795	H113
175	TH-538	ZSI_CDT_TH-538	*Thrips flavus*	BOLD:AAN6624	KX622344	6-Jun-14	31.942	77.1747	H115
176	TH-539	ZSI_CDT_TH-539	*Thrips flavus*	BOLD:AAN6624	KX622345	6-Jun-14	31.942	77.1747	H113
177	TH-314B	ZSI_CDT_TH-314B	*Thrips flavus*	BOLD:AAN6624	KX622346	11-Oct-13	32.5507	76.0188	H116
178	TH-999	ZSI_CDT_TH-999	*Thrips florum*	BOLD:AAP7681	KX622347	29-Dec-14	24.885	74.3338	H117
179	TH-785	ZSI_CDT_TH-785	*Thrips florum*	BOLD:AAP7681	KX622348	6-Dec-14	22.5667	88.3667	H117
180	TH-1A	ZSI_CDT_TH-1A	*Thrips florum*	BOLD:AAP7681	KF840094	22-Nov-11	31.1048	77.1734	H117
181	TH-3	ZSI_CDT_TH-3	*Thrips florum*	BOLD:AAP7681	KF840095	22-Nov-11	31.1048	77.1734	H117
182	TH-291A	ZSI_CDT_TH-291A	*Thrips florum*	BOLD:AAP7681	KX622349	10-Apr-14	22.51	88.334	H117
183	TH-1	ZSI_CDT_TH-1	*Thrips florum*	BOLD:AAP7681	KF643007	22-Nov-12	32.0196	76.1531	H117
184	TH-382	ZSI_CDT_TH-382	*Thrips florum*	BOLD:AAP7681	KX622350	15-Oct-13	32.139	76.4534	H117
185	TH-302	ZSI_CDT_TH-302	*Thrips florum*	BOLD:AAP7681	KX622351	10-Oct-13	32.539	75.9704	H117
186	TH-986	ZSI_CDT_TH-986	*Thrips hawaiiensis*	BOLD:AAZ8516	KX622352	29-Dec-14	24.6727	73.8775	H118
187	TH-286	ZSI_CDT_TH-286	*Thrips hawaiiensis*	BOLD:AAZ8516	KX622353	5-Mar-14	12.9716	77.5946	H118
188	TH-552	ZSI_CDT_TH-552	*Thrips hawaiiensis*	BOLD:AAZ8516	KX622354	6-Mar-14	23.3441	85.3096	H119
189	TH-92A	ZSI_CDT_TH-92A	*Thrips hawaiiensis*	BOLD:AAZ8516	KX622355	21-Mar-13	22.5726	88.3639	H118
190	TH-763	ZSI_CDT_TH-763	*Thrips hawaiiensis*	BOLD:AAZ8516	KX622356	1-Nov-14	23.1645	92.9376	H120
191	TH-554	ZSI_CDT_TH-554	*Thrips hawaiiensis*	BOLD:AAZ8516	KX622357	2-Nov-14	23.3441	85.3096	H121
192	TH-571	ZSI_CDT_TH-571	*Thrips hawaiiensis*	BOLD:AAZ8516	KX622358	1-Aug-14	11.4916	76.7337	H118
193	TH-553	ZSI_CDT_TH-553	*Thrips hawaiiensis*	BOLD:AAZ8516	KX622359	2-Aug-14	23.3441	85.3096	H118
194	TH-92	ZSI_CDT_TH-92	*Thrips hawaiiensis*	BOLD:AAZ8516	KF840096	21-Mar-13	22.5008	88.3681	H118
195	TH-290A	ZSI_CDT_TH-290A	*Thrips hawaiiensis*	BOLD:AAZ8516	KX622360	31-Mar-13	22.51	88.334	H118
196	TH-608A	ZSI_CDT_TH-608A	*Thrips moundi*	BOLD:ACY7915	KX622361	12-Jun-14	31.0583	77.2719	H122
197	TH-639	ZSI_CDT_TH-639	*Thrips moundi*	BOLD:ACY7915	KP993174	13-Jun-14	31.2109	77.3866	H123
198	TH-641	ZSI_CDT_TH-641	*Thrips moundi*	BOLD:ACY7915	KX622362	12-Jun-14	31.0522	77.2684	H124
199	TH-642	ZSI_CDT_TH-642	*Thrips moundi*	BOLD:ACY7915	KX622363	12-Jun-14	31.0522	77.2684	H122
200	TH-608B	ZSI_CDT_TH-608B	*Thrips moundi*	BOLD:ACY7915	KX622364	12-Jun-14	31.0583	77.2719	H122
201	TH-612	ZSI_CDT_TH-612	*Thrips moundi*	BOLD:ACY7915	KP993173	13-Jun-14	31.2109	77.3866	H122
202	TH-540	ZSI_CDT_TH-540	*Thrips orientalis*	BOLD:ACP2570	KM507077	1-Jul-14	16.8093	81.9917	H125
203	TH-541	ZS-_CDT_TH-541	*Thrips orientalis*	BOLD:ACP2570	KM507078	1-Jul-14	16.8093	81.9917	H125
204	TH-1007	ZSI_CDT_TH-1007	*Thrips palmi*	BOLD:AAE7913	KX622365	6-Apr-15	22.5602	88.4421	H126
205	TH-1062A	ZSI_CDT_TH-1062A	*Thrips palmi*	BOLD:AAE7913	KX622366	30-Dec-14	24.7166	73.6387	H126
206	TH-226	ZSI_CDT_TH-226	*Thrips palmi*	BOLD:AAE7913	KX622367	13-Jan-14	22.5406	88.3958	H126

207	TH-230	ZSI_CDT_TH-230	*Thrips palmi*	BOLD:AAE7913	KX622368	13-Jan-14	22.5406	88.3958	H126
208	TH-240	ZSI_CDT_TH-240	*Thrips palmi*	BOLD:AAE7913	KX622369	20-Jan-14	22.51	88.334	H126
209	TH-813	ZSI_CDT_TH-813	*Thrips palmi*	BOLD:AAE7913	KX622370	30-Dec-14	24.6511	73.651	H127
210	TH-1115	ZSI_CDT_TH-1115	*Thrips palmi*	BOLD:AAE7913	KX622371	16-Nov-15	21.85	84.03	H126
211	TH-1114	ZSI_CDT_TH-1114	*Thrips palmi*	BOLD:AAE7913	KX622372	16-Nov-15	21.85	84.03	H126
212	TH-1113	ZSI_CDT_TH-1113	*Thrips palmi*	BOLD:AAE7913	KX622373	16-Nov-15	21.85	84.03	H126
213	TH-1112	ZSI_CDT_TH-1112	*Thrips palmi*	BOLD:AAE7913	KX622374	16-Nov-15	21.85	84.03	H126
214	TH-1111	ZSI_CDT_TH-1111	*Thrips palmi*	BOLD:AAE7913	KX622375	16-Nov-15	21.85	84.03	H126
215	TH-1110	ZSI_CDT_TH-1110	*Thrips palmi*	BOLD:AAE7913	KX622376	16-Nov-15	21.85	84.03	H126
216	TH-1108	ZSI_CDT_TH-1108	*Thrips palmi*	BOLD:AAE7913	KX622377	16-Nov-15	21.85	84.03	H126
217	TH-1105	ZSI_CDT_TH-1105	*Thrips palmi*	BOLD:AAE7913	KX622378	14-Nov-15	21.85	84.03	H126
218	TH-877	ZSI_CDT_TH-877	*Thrips palmi*	BOLD:AAE7913	KX622379	6-Apr-15	22.5602	88.4421	H126
219	TH-1104	ZSI_CDT_TH-1104	*Thrips palmi*	BOLD:ACY9134	KX622380	14-Nov-15	21.85	84.03	H128
220	TH-1103	ZSI_CDT_TH-1103	*Thrips palmi*	BOLD:ACY9134	KX622381	14-Nov-15	21.85	84.03	H128
221	TH-971	ZSI_CDT_TH-971	*Thrips palmi*	BOLD:AAE7913	KX622382	1-Jun-15	20.2705	85.8064	H126
222	TH-997	ZSI_CDT_TH-997	*Thrips palmi*	BOLD:AAE7913	KX622383	6-Apr-15	22.5602	88.4421	H126
223	TH-879	ZSI_CDT_TH-879	*Thrips palmi*	BOLD:AAE7913	KX622384	6-Apr-15	22.5602	88.4421	H126
224	TH-878	ZSI_CDT_TH-878	*Thrips palmi*	BOLD:AAE7913	KX622385	6-Apr-15	22.5602	88.4421	H126
225	TH-875	ZSI_CDT_TH-875	*Thrips palmi*	BOLD:AAE7913	KX622386	6-Apr-15	22.5602	88.4421	H126
226	TH-868	ZSI_CDT_TH-868	*Thrips palmi*	BOLD:AAE7913	KX622387	25-Dec-14	26.2834	74.7516	H126
227	TH-1101	ZSI_CDT_TH-1101	*Thrips palmi*	BOLD:AAE7913	KX622388	14-Nov-15	21.85	84.03	H126
228	TH-1100	ZSI_CDT_TH-1100	*Thrips palmi*	BOLD:ACY9134	KX622389	14-Nov-15	21.85	84.03	H128
229	TH-1099	ZSI_CDT_TH-1099	*Thrips palmi*	BOLD:AAE7913	KX622390	14-Nov-15	21.85	84.03	H126
230	TH-1098	ZSI_CDT_TH-1098	*Thrips palmi*	BOLD:AAE7913	KX622391	14-Nov-15	21.85	84.03	H126
231	TH-1097	ZSI_CDT_TH-1097	*Thrips palmi*	BOLD:AAE7913	KX622392	14-Nov-15	21.85	84.03	H126
232	TH-1095	ZSI_CDT_TH-1095	*Thrips palmi*	BOLD:AAE7913	KX622393	14-Nov-15	21.85	84.03	H126
233	TH-226A	ZSI_CDT_TH-226A	*Thrips palmi*	BOLD:AAE7913	KX622394	13-Jan-14	22.5406	88.3958	H126
234	TH-230A	ZSI_CDT_TH-230A	*Thrips palmi*	BOLD:AAE7913	KX622395	13-Jan-14	22.5406	88.3958	H126
235	TH-770	ZSI_CDT_TH-770	*Thrips palmi*	BOLD:AAE7913	KX622396	15-Jun-14	30.5912	77.4889	H129
236	TH-868A	ZSI_CDT_TH-868A	*Thrips palmi*	BOLD:AAE7913	KX622397	25-Dec-14	26.2834	74.7516	H126
237	TH-873	ZSI_CDT_TH-873	*Thrips palmi*	BOLD:AAE7913	KX622398	6-Apr-15	22.5602	88.4421	H126
238	TH-1086	ZSI_CDT_TH-1086	*Thrips palmi*	BOLD:AAE7913	KX622399	16-Nov-15	21.85	84.03	H126
239	TH-1085	ZSI_CDT_TH-1085	*Thrips palmi*	BOLD:AAE7913	KX622400	16-Nov-15	21.85	84.03	H126
240	TH-1083	ZSI_CDT_TH-1083	*Thrips palmi*	BOLD:AAE7913	KX622401	16-Nov-15	21.85	84.03	H130
241	TH-1082	ZSI_CDT_TH-1082	*Thrips palmi*	BOLD:AAE7913	KX622402	16-Nov-15	21.85	84.03	H131
242	TH-874	ZSI_CDT_TH-874	*Thrips palmi*	BOLD:AAE7913	KX622403	6-Apr-15	22.5602	88.4421	H126
243	TH-1008	ZSI_CDT_TH-1008	*Thrips palmi*	BOLD:AAE7913	KX622404	6-Apr-15	22.5602	88.4421	H126
244	TH-1009	ZSI_CDT_TH-1009	*Thrips palmi*	BOLD:AAE7913	KX622405	6-Apr-15	22.5602	88.4421	H126
245	TH-1010	ZSI_CDT_TH-1010	*Thrips palmi*	BOLD:AAE7913	KX622406	6-Apr-15	22.5602	88.4421	H126
246	TH-1011	ZSI_CDT_TH-1011	*Thrips palmi*	BOLD:AAE7913	KX622407	6-Apr-15	22.5602	88.4421	H126
247	TH-1012	ZSI_CDT_TH-1012	*Thrips palmi*	BOLD:AAE7913	KX622408	6-Apr-15	22.5602	88.4421	H126
248	TH-1016	ZSI_CDT_TH-1016	*Thrips palmi*	BOLD:AAE7913	KX622409	6-Apr-15	22.5602	88.4421	H126
249	TH-1017	ZSI_CDT_TH-1017	*Thrips palmi*	BOLD:AAE7913	KX622410	6-Apr-15	22.5602	88.4421	H126
250	TH-1018	ZSI_CDT_TH-1018	*Thrips palmi*	BOLD:AAE7913	KX622411	6-Apr-15	22.5602	88.4421	H126

251	TH-1019	ZSI_CDT_TH-1019	*Thrips palmi*	BOLD:AAE7913	KX622412	6-Apr-15	22.5602	88.4421	H126
252	TH-1020	ZSI_CDT_TH-1020	*Thrips palmi*	BOLD:AAE7913	KX622413	6-Apr-15	22.5602	88.4421	H126
253	TH-1021	ZSI_CDT_TH-1021	*Thrips palmi*	BOLD:AAE7913	KX622414	6-Apr-15	22.5602	88.4421	H126
254	TH-1033	ZSI_CDT_TH-1033	*Thrips palmi*	BOLD:AAE7913	KX622415	6-Apr-15	22.5602	88.4421	H126
255	TH-1034	ZSI_CDT_TH-1034	*Thrips palmi*	BOLD:AAE7913	KX622416	6-Apr-15	22.5602	88.4421	H126
256	TH-1035	ZSI_CDT_TH-1035	*Thrips palmi*	BOLD:AAE7913	KX622417	6-Apr-15	22.5602	88.4421	H126
257	TH-1037	ZSI_CDT_TH-1037	*Thrips palmi*	BOLD:AAN2747	KX622418	2-Jun-15	20.2704	85.8063	H9
258	TH-1038	ZSI_CDT_TH-1038	*Thrips palmi*	BOLD:AAE7913	KX622419	13-Jan-14	22.5602	88.4421	H126
259	TH-1039	ZSI_CDT_TH-1039	*Thrips palmi*	BOLD:AAE7913	KX622420	13-Jan-14	22.5602	88.4421	H126
260	TH-1040	ZSI_CDT_TH-1040	*Thrips palmi*	BOLD:AAE7913	KX622421	13-Jan-14	22.5602	88.4421	H126
261	TH-1041	ZSI_CDT_TH-1041	*Thrips palmi*	BOLD:AAE7913	KX622422	13-Jan-14	22.5602	88.4421	H126
262	TH-1042	ZSI_CDT_TH-1042	*Thrips palmi*	BOLD:AAE7913	KX622423	13-Jan-14	22.5602	88.4421	H126
263	TH-1043	ZSI_CDT_TH-1043	*Thrips palmi*	BOLD:AAE7913	KX622424	13-Jan-14	22.5602	88.4421	H126
264	TH-1044	ZSI_CDT_TH-1044	*Thrips palmi*	BOLD:AAE7913	KX622425	13-Jan-14	22.5602	88.4421	H126
265	TH-72	ZSI_CDT_TH-72	*Thrips palmi*	BOLD:AAE7913	KF840097	5-Mar-13	22.5726	88.3639	H126
266	TH-1045	ZSI_CDT_TH-1045	*Thrips palmi*	BOLD:AAE7913	KX622426	13-Jan-14	22.5602	88.334	H126
267	TH-1046	ZSI_CDT_TH-1046	*Thrips palmi*	BOLD:AAE7913	KX622427	13-Jan-14	22.5602	88.4421	H126
268	TH-1047	ZSI_CDT_TH-1047	*Thrips palmi*	BOLD:AAE7913	KX622428	13-Jan-14	22.5602	88.4421	H126
269	TH-1062	ZSI_CDT_TH-1062	*Thrips palmi*	BOLD:AAE7913	KX622429	30-Dec-14	24.7166	73.6387	H126
270	TH-1048	ZSI_CDT_TH-1048	*Thrips palmi*	BOLD:AAE7913	KX622430	13-Jan-14	22.5602	88.4421	H126
271	TH-1002	ZSI_CDT_TH-1002	*Thrips palmi*	BOLD:AAE7913	KX622431	29-Dec-14	24.6727	73.8775	H126
272	TH-1049	ZSI_CDT_TH-1049	*Thrips palmi*	BOLD:AAE7913	KX622432	13-Jan-14	22.5602	88.4421	H126
273	TH-1050	ZSI_CDT_TH-1050	*Thrips palmi*	BOLD:AAE7913	KX622433	13-Jan-14	22.5602	88.4421	H126
274	TH-1052	ZSI_CDT_TH-1052	*Thrips palmi*	BOLD:AAE7913	KX622434	13-Jan-14	22.5602	88.4421	H126
275	TH-1053	ZSI_CDT_TH-1053	*Thrips palmi*	BOLD:AAE7913	KX622435	13-Jan-14	22.5602	88.4421	H126
276	TH-1054	ZSI_CDT_TH-1054	*Thrips palmi*	BOLD:AAE7913	KX622436	13-Jan-14	22.5602	88.4421	H126
277	TH-1055	ZSI_CDT_TH-1055	*Thrips palmi*	BOLD:AAE7913	KX622437	13-Jan-14	22.5602	88.4421	H126
278	TH-276C	ZSI_CDT_TH-276C	*Thrips parvispinus*	BOLD:AAM8085	KM485661	10-Feb-14	13.132	77.4893	H132
279	TH-283B	ZSI_CDT_TH-283B	*Thrips parvispinus*	BOLD:AAM8085	KM485665	10-Feb-14	13.132	77.4893	H132
280	TH-427B	ZSI_CDT_TH-427B	*Thrips parvispinus*	BOLD:AAM8085	KM485667	10-Feb-14	13.132	77.4893	H132
281	TH-283A	ZSI_CDT_TH-283A	*Thrips parvispinus*	BOLD:AAM8085	KM485664	10-Feb-14	13.132	77.4893	H132
282	TH-277B	ZSI_CDT_TH-277B	*Thrips parvispinus*	BOLD:AAM8085	KM485663	10-Feb-14	12.9667	77.5667	H132
283	TH-276A	ZSI_CDT_TH-276A	*Thrips parvispinus*	BOLD:AAM8085	KM485659	10-Feb-14	13.132	77.4893	H132
284	TH-427A	ZSI_CDT_TH-427A	*Thrips parvispinus*	BOLD:AAM8085	KM485666	10-Feb-14	13.132	77.4893	H132
285	TH-276B	ZSI_CDT_TH-276B	*Thrips parvispinus*	BOLD:AAM8085	KM485660	10-Feb-14	13.132	77.4893	H132
286	TH-277A	ZSI_CDT_TH-277A	*Thrips parvispinus*	BOLD:AAM8085	KM485662	10-Feb-14	12.9667	77.5667	H132
287	TH-1026	ZSI_CDT_TH-1026	*Thrips subnudula*	BOLD:ACY8465	KX622438	2-Jun-15	20.2857	85.7916	H133
288	TH-1023	ZSI_CDT_TH-1023	*Thrips subnudula*	BOLD:ACY8465	KX622439	2-Jun-15	20.2857	85.7916	H133
289	TH-1024	ZSI_CDT_TH-1024	*Thrips subnudula*	BOLD:ACY8465	KX622440	2-Jun-15	20.2857	85.7916	H133
290	TH-1025	ZSI_CDT_TH-1025	*Thrips subnudula*	BOLD:ACY8465	KX622441	2-Jun-15	20.2857	85.7916	H133
291	TH-970	ZSI_CDT_TH-970	*Thrips subnudula*	BOLD:ACY8465	KX622442	1-Jun-15	20.2705	85.8064	H133
292	TH-508	ZSI_CDT_TH-508	*Thrips tabaci*	BOLD:AAB3870	KX622443	2-Jun-15	15.3173	75.7139	H134
293	TH-533	ZSI_CDT_TH-533	*Thrips tabaci*	BOLD:AAB3870	KX622444	6-Jun-14	31.942	77.1747	H11
294	TH-1116	ZSI_CDT_TH-1116	*Thrips tabaci*	BOLD:AAB3870	KX622445	25-Dec-14	26.2833	74.7516	H11

295	TH-140	ZSI_CDT_TH-140	*Thrips tabaci*	BOLD:AAB3870	KX622446	5-Jan-13	16.155	76.5199	H135
296	TH-139	ZSI_CDT_TH-139	*Thrips tabaci*	BOLD:AAB3870	KX622447	26-Sep-12	16.212	77.3439	H135
297	TH-1077	ZSI_CDT_TH-1077	*Tusothrips sumatrensis*	BOLD:ACY9997	KX622448	1-Jun-15	20.2704	85.8063	H136
298	TH-80	ZSI_CDT_TH-80	*Androthrips flavipes*	BOLD:ACY7674	KX622187	14-Mar-13	17.37	78.48	H21
299	TH-916	ZSI_CDT_TH-916	*Arrhenothrips acuminatus*	BOLD:ACY8495	KX622190	13-May-15	22.5418	88.3622	H23
300	TH-913	ZSI_CDT_TH-913	*Arrhenothrips acuminatus*	BOLD:ACY8495	KX622191	13-May-15	22.5418	88.3622	H23
301	TH-912	ZSI_CDT_TH-912	*Arrhenothrips acuminatus*	BOLD:ACY8495	KX622192	13-May-15	22.5418	88.3622	H23
302	TH-914	ZSI_CDT_TH-914	*Arrhenothrips acuminatus*	BOLD:ACY8495	KX622193	13-May-15	22.5418	88.3622	H23
303	TH-915	ZSI_CDT_TH-915	*Arrhenothrips acuminatus*	BOLD:ACY8495	KX622194	13-May-15	22.5418	88.3622	H23
304	TH-90	ZSI_CDT_TH-90	*Azaleothrips amabilis*	BOLD:ACY8434	KX622197	21-Mar-13	22.34	88.22	H27
305	TH-850	ZSI_CDT_TH-850	*Dolichothrips citripes*	BOLD:ACV7278	KX622211	22-Mar-13	22.54	88.36	H35
306	TH-851	ZSI_CDT_TH-851	*Dolichothrips citripes*	BOLD:ACV7278	KX622212	23-Mar-13	22.34	88.22	H35
307	TH-1117A	ZSI_CDT_TH-1117A	*Dolichothrips ochripes*	BOLD:ACY9910	KX622213	25-Dec-14	26.2833	74.7516	H36
308	TH-1117	ZSI_CDT_TH-1117	*Dolichothrips ochripes*	BOLD:ACY9910	KX622214	25-Dec-14	26.2833	74.7516	H37
309	TH-795	ZSI_CDT_TH-795	*Dyothrips sp.*	BOLD:ACY7630	KX622215	21-Dec-14	26.7512	75.9357	H38
310	TH-297	ZSI_CDT_TH-297	*Gynaikothrips uzeli*	BOLD:AAM8067	KX622229	10-Apr-13	22.51	88.334	H47
311	TH-146	ZSI_CDT_TH-146	*Gynaikothrips uzeli*	BOLD:AAM8067	KX622230	10-Jul-13	22.5726	88.3639	H48
312	TH-113	ZSI_CDT_TH-113	*Gynaikothrips uzeli*	BOLD:AAM8067	KF840085	7-May-13	22.5415	88.35	H47
313	TH-81	ZSI_CDT_TH-81	*Gynaikothrips uzeli*	BOLD:AAM8067	KX622231	11-Jul-13	15.9129	79.74	H47
314	TH-77	ZSI_CDT_TH-77	*Haplothrips andresi*	BOLD:AAN5799	KF840086	26-Feb-13	22.51	88.334	H49
315	TH-941	ZSI_CDT_TH-941	*Haplothrips bagrolis*	BOLD:AAT9984	KX622232	1-Jun-15	20.2704	85.8063	H50
316	TH-105A	ZSI_CDT_TH-105A	*Haplothrips ceylonicus*	BOLD:ACY8731	KX622233	1-Mar-13	22.51	88.334	H51
317	TH-105B	ZSI_CDT_TH-105B	*Haplothrips ceylonicus*	BOLD:ACY8730	KX622234	1-Mar-13	22.51	88.334	H51
318	TH-937	ZSI_CDT_TH-937	*Haplothrips ganglbaueri*	BOLD:ACF1370	KX622235	28-Dec-14	24.5691	73.7005	H52
319	TH-969	ZSI_CDT_TH-969	*Haplothrips ganglbaueri*	BOLD:ACF1370	KX622236	27-Dec-14	25.5013	73.8513	H52
320	TH-938	ZSI_CDT_TH-938	*Haplothrips ganglbaueri*	BOLD:ACF1370	KX622237	28-Dec-14	24.5686	73.818	H52
321	TH-823	ZSI_CDT_TH-823	*Haplothrips ganglbaueri*	BOLD:ACF1370	KX622238	21-Dec-14	26.7512	75.9357	H52
322	TH-812	ZSI_CDT_TH-812	*Haplothrips ganglbaueri*	BOLD:ACF1370	KX622239	30-Dec-14	24.6511	73.651	H52
323	TH-794	ZSI_CDT_TH-794	*Haplothrips ganglbaueri*	BOLD:ACF1370	KX622240	21-Dec-14	26.7512	75.9357	H52
324	TH-942	ZSI_CDT_TH-942	*Haplothrips gowdeyi*	BOLD:ACZ0203	KX622241	1-Jun-15	20.2704	85.8063	H53
325	TH-414	ZSI_CDT_TH-414	*Haplothrips reuteri*	BOLD:AAI6863	KX622242	4-Jun-14	31.427	77.0746	H54
326	TH-74	ZSI_CDT_TH-74	*Xylaplothrips pusillus*	BOLD:ACI5935	KF840087	14-Feb-13	12.77	75.22	H55
327	TH-783	ZSI_CDT_TH-783	*Xylaplothrips pusillus*	BOLD:ACI5935	KX622243	7-Dec-14	22.5667	88.3667	H56
328	TH-388B	ZSI_CDT_TH-388B	*Haplothrips tenuipennis*	BOLD:AAN4488	KX622244	8-Dec-14	31.9731	76.8489	H57
329	TH-476	ZSI_CDT_TH-476	*Haplothrips tenuipennis*	BOLD:AAN4488	KX622245	5-Jun-14	31.4342	77.0718	H57
330	TH-388A	ZSI_CDT_TH-388A	*Haplothrips tenuipennis*	BOLD:AAN4488	KX622246	15-Oct-13	31.9731	76.8489	H57
331	TH-968	ZSI_CDT_TH-968	*Idiothrips ficus*	BOLD:ACY9602	KX622252	27-Dec-14	25.5013	73.8513	H62
332	TH-1094	ZSI_CDT_TH-1094	Phlaeothripidae	BOLD:ACZ0334	KX622278	2-Jan-15	26.2356	72.9857	H78
333	TH-797	ZSI_CDT_TH-797	*Plicothrips apicalis*	BOLD:AAN6622	KX622279	29-Dec-14	24.7346	73.9191	H79
334	TH-944	ZSI_CDT_TH-944	*Plicothrips apicalis*	BOLD:AAN6622	KX622280	1-Jun-15	20.2704	85.8063	H79
335	TH-945	ZSI_CDT_TH-945	*Plicothrips apicalis*	BOLD:AAN6622	KX622281	1-Jun-15	20.2704	85.8063	H79
336	TH-796	ZSI_CDT_TH-796	*Plicothrips apicalis*	BOLD:AAN6622	KX622282	21-Dec-14	26.7512	75.9357	H79

Table S2: Acquired GenBank accession numbers, Species name and estimated haplotypes included the study.

Sl No.	Gen Bank Acc. No.	Species	Haplotype
1	KF606954	*Franklinothrips vespiformis*	H1
2	KF606953	*Anaphothrips sudanensis*	H2
3	KF606951	*Frankliniella schultzei*	H3
4	KF606952	*Frankliniella schultzei*	H3
5	KF606950	*Frankliniella schultzei*	H3
6	Kf606949	*Frankliniella schultzei*	H3
7	KF015511	*Megalurothrips usitatus*	H4
8	KF015513	*Megalurothrips usitatus*	H4
9	KF015512	*Megalurothrips usitatus*	H4
10	HQ230353	*Sciothrips cardomomi*	H5
11	HQ230351	*Sciothrips cardomomi*	H5
12	HQ230349	*Sciothrips cardomomi*	H5
13	HQ230352	*Sciothrips cardomomi*	H6
14	HQ230350	*Sciothrips cardomomi*	H7
15	KF015507	*Scirtothrips dorsalis*	H8
16	HQ377270	*Scirtothrips dorsalis*	H8
17	KF015510	*Scirtothrips dorsalis*	H8
18	KF015509	*Scirtothrips dorsalis*	H8
19	KF015506	*Scirtothrips dorsalis*	H8
20	KF015508	*Scirtothrips dorsalis*	H8
21	KF015500	*Thrips palmi*	H9
22	KF015503	*Thrips palmi*	H9
23	KF015505	*Thrips palmi*	H9
24	KF015496	*Thrips palmi*	H9
25	KF015499	*Thrips palmi*	H9
26	KF015498	*Thrips palmi*	H9
27	HQ377269	*Thrips palmi*	H9
28	KF015501	*Thrips palmi*	H9
29	KF015504	*Thrips palmi*	H9
30	KF015497	*Thrips palmi*	H9
31	KF015502	*Thrips palmi*	H10
32	KF015434	*Thrips tabaci*	H11
33	KF015432	*Thrips tabaci*	H11
34	KF015433	*Thrips tabaci*	H11

Table S3: Nomenclature of Molecular Operational Taxonomic Units (MOTUs) estimated by superimposing results of four automatic delimitation methods (ABGD, GMYC, BIN, and bPTP) for all studied species.

Sl No.	Species	Sample ID	Nomenclature
1.	*Aduncothrips asiaticus*	TH-929, TH-73	Ia1
2.	*Aeolothrips distinctus*	TH-232	Ia1
		TH-383, TH-384, TH-342	IIa1
3.	*Aeolothrips intermedius*	TH-832	Ia1
		TH-853	IIa1
4.	*Franklinothrips megalops*	TH-1074	Ia1
		TH-189, TH-185, TH-187	IIa1
5.	*Franklinothrips vespiformis*	TH-989, TH-1003, TH-1088, TH-58A, TH-58, TH-991	Ia1
6.	*Mymarothrips garuda*	TH-927, TH-270, TH-928	Ia1
7.	*Streothrips sp.*	TH-990	Ia1
8.	*Holarthrothrips indicus*	TH-811, TH-825	Ia1
9.	*Astrothrips stannardi*	TH-70	Ia1
10.	*Astrothrips tumiceps*	TH-988	Ia1

11.	*Caliothrips indicus*	TH-1092, TH-1066, TH-931, TH-1067, TH-831	Ia1
12.	*Helionothrips parvus*	TH-618	Ia1
13.	*Helionothrips aino*	TH-431	Ia1
14.	*Heliothrips haemorrhoidalis*	TH-336	Ia1
15.	*Phibalothrips peringueyi*	TH-624, TH-631, TH-630	Ia1
16.	*Retithrips syriacus*	TH-996, TH-780	Ia1
17.	*Rhipiphorothrips cruentatus*	TH-995	Ia1
18.	*Selenothrips rubrocinctus*	TH-76	Ia1
19.	*Neohydatothrips chandrai*	TH-1079	Ia1
20.	*Neohydatothrips plumeria*	TH-1123	Ia1
21.	*Neohydatothrips samayunkur*	TH-341, TH-939, TH-1001, TH-309, TH-1000	Ia1
22.	*Anaphothrips obscurus*	TH-317A, TH-587A	Ia1
23.	*Anaphothrips sudanensis*	TH-314A, TH-1121, TH-1073, TH-817, TH-586, TH-581A, TH-816, TH-312, KF606953	Ia1
24.	*Aptinothrips rufus*	TH-313A, TH-585A	Ia1
25.	*Ayyaria chaetophora*	TH-930	Ia1
26.	*Bathrips melanicornis*	TH-65, TH-1075, TH-197, TH-195, TH-196	Ia1
27.	*Biltothrips minutus*	TH-224, TH-269, TH-268, TH-223	Ia1
28.	*Bolacothrips striatopennatus*	TH-820, TH-790	Ia1
29.	*Chirothrips africanus*	TH-815, TH-1028A, TH-818	Ia1
30.	*Diarthrothrips nimbus*	TH-1032	Ia1
31.	*Dendrothripoides innoxius*	TH-936	Ia1
32.	*Eremiothrips antilope*	TH-1091, TH-1093, TH-1014, TH-1015, TH-1090	Ia1
33.	*Exothrips ornus*	TH-791	Ia1
34.	*Florithrips traegardhi*	TH-821, TH-822	Ia1
35.	*Frankliniella intonsa*	TH-84	Ia1
36.	*Frankliniella schultzei*	TH-807, TH-808, TH-809	Ia1
		TH-1080, TH-1031, TH-923	IIa1
		KF605949, KF605950, KF605951, KF605952	IIIa1
37.	*Frankliniella unicolor*	TH-452 , TH-451B, TH-451A	Ia1
38.	*Lefroyothrips lefroyi*	TH-7, TH-378, TH-379, TH-8	Ia1
39.	*Megalurothrips usitatus*	KF015511, KF015512, KF015513	Ia1
40.	*Megalurothrips typicus*	TH-762, TH-833	Ia1
41.	*Microcephalothrips abdominalis*	TH-580, TH-1122, TH-935	Ia1
42.	*Moundinothrips robustus*	TH-617	Ia1
43.	*Mycterothrips nilgiriensis*	TH-599	Ia1
		TH-610, TH-14, TH-438	IIa1
44.	*Oxythrips kochummani*	TH-566B, TH-306B	Ia1
45.	*Parabaliothrips takahashii*	TH-615	Ia1
46.	*Sciothrips cardamomi*	HQ230349, HQ230350, HQ230351, HQ230352, HQ230353	Ia1
47.	*Scirtothrips dorsalis*	TH-972, TH-861, TH-934, TH-857 KF015507, HQ377270, KF015509,KF015506, KF015508	Ia1
48.	*Scirtothrips sp.*	TH-477	Ia1
49.	*Scirtothrips oligochaetus*	TH-1087	Ia1
		TH-983, TH-1118, TH-834	IIa1
50.	*Scirtothrips kenyensis*	TH-640	Ia1
51.	*Smilothrips productus*	TH-632, TH-621, TH-623, TH-566A	Ia1
52.	*Stenchaetothrips biformis*	TH-987	Ia1
53.	*Stenchaetothrips pteratus*	TH-310B, TH-310A	Ia1
54.	*Stenchaetothrips spinulae*	TH-71, TH-271, TH-274, TH-273, TH-275	Ia1
55.	*Taeniothrips bharokariiensis*	TH-434A, TH-434B, TH-435	Ia1
56.	*Tenothrips frici*	TH-979, TH-978	Ia1
57.	*Thrips alatus*	TH-441	Ia1
		TH-327	IIa1
58.	*Thrips andrewsi*	TH-542, TH-536, TH-535, TH-418, TH-534	Ia1
		TH-547, TH-546, TH-545, TH-544, TH-543	IIa1
59.	*Thrips apicatus*	TH-1006, TH-557	Ia1
		TH-1119, TH-1120, TH-904, TH-984	IIa1
60.	*Thrips carthami*	TH-386	Ia1
		TH-587B, TH-317B, TH-325, TH-318, TH-316,TH-320	Ia2
61.	*Thrips coloratus*	TH-528	Ia1

62.	*Thrips flavus*	TH-548, TH-313B, TH-585B, TH-428, TH-532, TH-537, TH-531, TH-530, TH-581B, TH-538, TH-539, TH-314B	Ia1
63.	*Thrips florum*	TH-999, TH-785, TH-1A, TH-3, TH-291A, TH-1, TH-382, TH-302	Ia1
64.	*Thrips hawaiiensis*	TH-986, TH-286, TH-552, TH-92A, TH-763, TH-554, TH-571, TH-553, TH-92, TH-290A	Ia1
65.	*Thrips moundi*	TH-608A, TH-639, TH-641, TH-642, TH-608B,TH-612	Ia1
		TH-641	Ia2
66.	*Thrips orientalis*	TH-540, TH-541	Ia1
67.	*Thrips palmi*	TH-1007, TH-1062A, TH-226, TH-230, TH-240, TH-813, TH-1115, TH-1114, TH-1113, TH-1112, TH-1111, TH-1110, TH-1108, TH-1105, TH-877, TH-971, TH-997, TH-879, TH-878, TH-875, TH-868, TH-1101, TH-1099, TH-1098, TH-1097, TH-1095, TH-226A, TH-230A, TH-770, TH-868A, TH-873, TH-1086, TH-1085, TH-1083, TH-1082, TH-874, TH-1008, TH-1009, TH-1010, TH-1011, TH-1012, TH-1016, TH-1017, TH-1018, TH-1019, TH-1020, TH-1021, TH-1033, TH-1034, TH-1035, TH-1038, TH-1039, TH-1040, TH-1041, TH-1042, TH-1043, TH-1044, TH-72, TH-1045, TH-1046, TH-1047, TH-1062, TH-1048, TH-1002, TH-1049, TH-1050, TH-1052, TH-1053, TH-1054, TH-1055	Ia1
		TH-1100, TH-1104, TH-1103	IIa1
		TH-1037	Ib1
		KF015496, KF015497, KF015498, KF015499, KF015500, KF015503, KF015504, KF015505, KF015507, HQ377269	Ib2
68.	*Thrips parvispinus*	TH-276C, TH-283B, TH-427B, TH-283A, TH-277B, TH-276A, TH-427A, TH-276B, TH-277A	Ia1
69.	*Thrips subnudula*	TH-1026, TH-970, TH-1023, TH-1024, TH-1025	Ia1
70.	*Thrips tabaci*	TH-533, TH-1116, TH-140, TH-139, KF15434, KF15432, KF15433, TH-508	Ia1
71.	*Tusothrips sumatrensis*	TH-1077	Ia1
72.	*Androthrips flavipes*	TH-80	Ia1
73.	*Arrhenothrips acuminatus*	TH-916, TH-913, TH-912, TH-914, TH-915	Ia1
74.	*Azaleothrips amabilis*	TH-90	Ia1
75.	*Dolichothrips citripes*	TH-850, TH-851	Ia1
76.	*Dolichothrips ochripes*	TH-1117A, TH-1117	Ia1
77.	*Dyothrips sp.*	TH-795	Ia1
78.	*Gynaikothrips uzeli*	TH-297, TH-146, TH-113, TH-81	Ia1
79.	*Haplothrips andresi*	TH-77	Ia1
80.	*Haplothrips bagrolis*	TH-941	Ia1
81.	*Haplothrips ceylonicus*	TH-105A	Ia1
		TH-105B	IIa1
82.	*Haplothrips ganglbaueri*	TH-937, TH-969, TH-938, TH-823, TH-812, TH-794	Ia1
83.	*Haplothrips gowdeyi*	TH-942	Ia1
84.	*Haplothrips reuteri*	TH-414	Ia1
85.	*Xylaplothrips pusillus*	TH-74	Ia1
		TH-783	IIa1
86.	*Haplothrips tenuipennis*	TH-388B, TH-476, TH-388A	Ia1
87.	*Idiothrips ficus*	TH-968	Ia1
88.	Phlaeothripidae	TH-1094	Ia1
89.	*Plicothrips apicalis*	TH-797, TH-944, TH-945, TH-796	Ia1

Table S4: K2P genetic distance of the 89 studied thrips species including acquired GenBank sequences.

	**Comparisons**	**Min**	**Mean**	**Max**	**SE**
Within Species	3224	0.00	0.0101	0.1830	0.0020
Within Genus	9510	0.0135	0.1871	0.2775	0.0066
Within Family	25329	0.103	0.2362	0.3797	0.0100

Table S5. Results of the Automatic Barcode Gap Discovery (ABGD) analyses. X, relative gap width; Simple, p-distance; Jukes-Cantor substitution model (JC69); Kimura 2-parameter substitution model (K2P).

Prior intraspecific distance (*P*)											
Model	X	Partition	0.0599	0.0359	0.0215	0.0129	0.0077	0.0046	0.0028	0.0017	0.0010
Simple	1.5	Initial	94	94	94	94	94	94	94	94	94
		Recursive	-	-	-	-	95	96	101	101	101
JC	1.5	Initial	94	94	94	94	94	94	94	94	202
		Recursive	-	-	-	98	101	111	112	112	-
K2P	1.5	Initial	94	94	94	94	94	94	94	94	202
		Recursive	-	-	-	98	101	111	113	113	-

Table S6. **Results of the General Mixed Yule-coalescent (GMYC) analyses.** Clusters, OTUs delineated by the GMYC model with multiple specimens; Entities, singleton OTUs delineated by GMYC; CI, confidence interval; Likelihood null, likelihood of the null model; Likelihood GMYC, likelihood of the GMYC model; Threshold, the threshold between the speciation and coalescence processes; Single, single-threshold model; Multiple, multiple-threshold model.

**Analysis**	**Clusters (CI)**	**Entities (CI)**	**Likelihood null**	**Likelihood GMYC**	**Likelihood ratio**	**Threshold**
Single	22 (19-24)	96 (93-107)	722.301	730.5517	16.50126	-0.02544052
Multiple	38 (23-38)	87 (85-93)	722.301	735.8739	27.14579	-0.217824<br>-0.1760951<br>-0.143071<br>-0.128906<br>-0.1017397<br>-0.06646273<br>-0.008443683<br>-0.003455731

Table S7: Detailed results of BINs from the "BIN Discordance Report" using BOLD. The BINs are sorted according to the rank of conflict to species level. The white, gray and yellow highlights represent discordance BINs, concordance BINs and singletons respectively.

**Identification**	**Conflicting Taxon in BIN**	**Rank of Conflict**	**BIN**	**BIN Total Members**	**BIN Tax Variation**
Mycterothrips nilgiriensis	*Mycterothrips*	Genus	BOLD:AAP7685	7	*Chaetanaphothrips*[4], *Mycterothrips*[3]
Neohydatothrips chandrai	*Neohydatothrips*	Genus	BOLD:ACG8261	2	*Pseudodendrothrips*[1], *Neohydatothrips*[1]
Aeolothrips distinctus	*Aeolothrips distinctus*	Species	BOLD:AAN6626	2	*Aeolothrips* PK01[1], *Aeolothrips distinctus*[1]
Dolichothrips citripes	*Dolichothrips citripes*	Species	BOLD:ACV7278	4	*Dolichothrips sp.*[2], *Dolichothrips citripes*[2]
Gynaikothrips uzeli	*Gynaikothrips uzeli*	Species	BOLD:AAM8067	43	*Gynaikothrips ficorum*[31], *Gynaikothrips uzeli*[12]
Haplothrips andresi	*Haplothrips andresi*	Species	BOLD:AAN5799	16	*Haplothrips andresi* [8], *Haplothrips tritici* [1]
Haplothrips bagrolis	*Haplothrips bagrolis*	Species	BOLD: AT9984	17	*Haplothrips ganglbaueri*[15], *Haplothrips bagrolis*[2]
Haplothrips ceylonicus	*Haplothrips ceylonicus*	Species	BOLD:ACY8730	8	*Haplothrips sp.*[1], *Haplothrips ceylonicus* [1]
Taeniothrips	*Taeniothrips*	Species	BOLD:ACP1595	6	*Taeniothrips sp.*[3], *Taeniothrips*

bharokariiensis	*bharokariiensis*				*bharokariiensis* [3]
Thrips flavus	*Thrips flavus*	Species	BOLD:AAN6624	128	*Thrips flavidulus* [116], *Thrips flavus*[12]
Thrips palmi	*Thrips palmi*	Species	BOLD:AAE7913	83	*Thrips palmi*[105], *Thrips tabaci*[1]
Thrips parvispinus	*Thrips parvispinus*	Species	BOLD:AAM8085	92	*Thrips parvispinus* [91], *Thrips palmi* [1]
Aduncothrips asiatcus		Species	BOLD:ACI5305	2	
Aeolothrips distinctus		Species	BOLD:ACY8456	3	
Aeolothrips intermedius		Species	BOLD:AAZ8618	12	
Anaphothrips obscurus		Species	BOLD:AAQ0558	47	
Anaphothrips sudanensis		Species	BOLD:AAV3388	11	
Aptinothrips rufus		Species	BOLD:ACY7882	2	
Arrhenothrips acuminatus		Species	BOLD:ACY8495	5	
Bathrips melanicornis		Species	BOLD:ACI6059	5	
Biltothrips minutus		Species	BOLD:ACL7167	4	
Bolacothrips striatopennatus		Species	BOLD:ACY8904	2	
Caliothrips indicus		Species	BOLD:ACY7911	5	
Chirothrips africanus		Species	BOLD:ACR6888	4	
Diarthrothrips nimbus		Species	BOLD:ACP4916	2	
Dolichothrips ochripes		Species	BOLD:ACY9910	2	
Eremiothrips antilope		Species	BOLD:ACZ0015	5	
Florithrips traegardhi		Species	BOLD:AAN9111	10	
Frankliniella intonsa		Species	BOLD:AAF6737	44	
Frankliniella schultzei		Species	BOLD:ACY9272	3	
Frankliniella schultzei		Species	BOLD:AAM8089	15	
Frankliniella unicolor		Species	BOLD:ACR6111	6	
Franklinothrips megalops		Species	BOLD:ACM2095	7	
Franklinothrips vespiformis		Species	BOLD:ACD8336	9	
Haplothrips ganglbaueri		Species	BOLD:ACF1370	45	
Haplothrips reuteri		Species	BOLD:AAI6863	2	
Xylaplothrips pusillus		Species	BOLD:ACI5935	3	
Haplothrips tenuipennis		Species	BOLD:AAN4488	28	
Heliothrips haemorrhoidalis		Species	BOLD:AAE5223	57	
Holarthrothrips indicus		Species	BOLD:ACY8375	2	
Lefroyothrips lefroyi		Species	BOLD:ACI6048	6	
Megalurothrips typicus		Species	BOLD:ACY7764	2	
Microcephalothrips abdominalis		Species	BOLD:AAI0410	39	
Moundinothrips robustus		Species	BOLD:ACR0282	2	
Mymarothrips garuda		Species	BOLD:ACL6651	3	
Neohydatothrips samayunkur		Species	BOLD:AAP7680	15	
Oxythrips kochummani		Species	BOLD:ACY8447	2	
Phibalothrips peringueyi		Species	BOLD:ACY8031	3	
Plicothrips apicalis		Species	BOLD:AAN6622	20	
Retithrips syriacus		Species	BOLD:ACY7969	2	
Scirtothrips dorsalis		Species	BOLD:ACV7644	167	
Scirtothrips oligochaetus		Species	BOLD:AAZ8518	5	
Smilothrips productus		Species	BOLD:ACY7742	4	
Stenchaetothrips pteratus		Species	BOLD:ACY8903	2	
Stenchaetothrips spinulae		Species	BOLD:ACI5207	5	
Tenothrips frici		Species	BOLD:ACY9167	2	
Thrips alatus		Species	BOLD:AAN6625	3	
Thrips andrewsi		Species	BOLD:ACA3048	13	
Thrips apicatus		Species	BOLD:AAY6262	9	
Thrips apicatus		Species	BOLD:ACY8630	2	
Thrips carthami		Species	BOLD:AAP7682	13	
Thrips coloratus		Species	BOLD:AAK1804	16	

Thrips florum		Species	BOLD:AAP7681	13	
Thrips hawaiiensis		Species	BOLD:AAZ8516	14	
Thrips moundi		Species	BOLD:ACY7915	8	
Thrips orientalis		Species	BOLD:ACP2570	4	
Thrips palmi		Species	BOLD:ACY9134	3	
Thrips palmi		Species	BOLD:AAN2747	112	
Thrips subnudula		Species	BOLD:ACY8465	5	
Thrips tabaci		Species	BOLD:AAB3870	316	
Phlaeothripidae		Family	BOLD:ACZ0334		
Androthrips flavipes		Species	BOLD:ACY7674		
Astrothrips stannardi		Species	BOLD:ACI7059		
Astrothrips tumiceps		Species	BOLD:ACY9954		
Ayyaria chaetophora		Species	BOLD:ACZ0116		
Azaleothrips amabilis		Species	BOLD:ACY8434		
Dendrothripoides innoxius		Species	BOLD:ACZ0271		
Dyothrips sp.		Species	BOLD:ACY7630		
Exothrips ornus		Species	BOLD:ACY8436		
Haplothrips ceylonicus		Species	BOLD:ACY8731		
Haplothrips gowdeyi		Species	BOLD:ACZ0203		
Helionothrips parvus		Species	BOLD:ACY8245		
Helionothrips aino		Species	BOLD:ACY8267		
Idiothrips ficus		Species	BOLD:ACY9602		
Mycterothrips nilgiriensis		Species	BOLD:ACY8466		
Neohydatothrips plumeria		Species	BOLD:ACZ0079		
Parabaliothrips takahashii		Species	BOLD:ACY7659		
Rhipiphorothrips cruentatus		Species	BOLD:ACY9953		
Scirtothrips kenyensis		Species	BOLD:ACY7741		
Scirtothrips sp.		Species	BOLD:ACY7963		
Selenothrips rubrocinctus		Species	BOLD:ACN6317		
Stenchaetothrips biformis		Species	BOLD:ACY9843		
Streothrips sp.		Species	BOLD:ACY9996		
Thrips carthami		Species	BOLD:ACY8520		
Tusothrips sumatrensis		Species	BOLD:ACY9997		
